# Development and design of the first structured clinic-based program in lower resource settings to transition emerging adults with type 1 diabetes from pediatric to adult care

**DOI:** 10.1371/journal.pgph.0000665

**Published:** 2022-08-03

**Authors:** Angelica Cristello Sarteau, Ariba Peerzada, Alpesh Goyal, Pradeep A. Praveen, Nikhil Tandon

**Affiliations:** 1 Department of Nutrition, University of North Carolina at Chapel Hill, Chapel Hill, North Carolina, United States of America; 2 Department of Endocrinology and Metabolism, All India Institute of Medical Sciences, Delhi, India; Brigham and Women’s Hospital, GUATEMALA

## Abstract

**Introduction:**

Type 1 diabetes (T1D) is increasing in young people worldwide and more children in resource limited settings are living into adulthood. There is a need for rigorous testing and reporting of evidence-based and stakeholder-informed strategies that transition individuals with T1D from pediatric to adult care. We present the development of and design of the first structured transition program in Delhi, India, to inform similar efforts in India and resource limited settings.

**Methods:**

The intervention development team included clinicians and researchers with expertise in T1D and the implementation context. To select intervention outcomes, establish intervention targets, and design session modules, we drew upon formative research conducted at prospective intervention implementation sites, consensus guidelines, and previous care transition and behavior change research conducted in developed settings. We used the Template for Intervention Description and Replication and GUIDance for the rEporting of intervention Development checklists to report the intervention and development process.

**Results:**

The 15-month program (“PATHWAY”) includes five quarterly ~30 minute sessions delivered predominantly by diabetes educators at pediatric and adult clinics, which coincide with routine care visits. Primary program components include educational and behavioral sessions that address psychosocial drivers of clinic attendance and self-management, diabetes educators as transition coordinators and counselors, and a one-year “overlap period” of alternating visits between pediatric and adult providers.

**Conclusions:**

We followed a systematic and transparent process to develop PATHWAY, which facilitated rich description of intervention context, guiding principles, targets, and components. Dependence on previously published program examples to design PATHWAY may have introduced challenges for program feasibility and effectiveness, underscoring the importance of input gathering from prospective intervention actors at multiple points in the development process. This detailed report in combination with future evaluations of PATHWAY support efforts to increase rigorous development and testing of strategies to improve outcomes among emerging adults with T1D.

## Introduction

One of the most common chronic illnesses diagnosed in childhood, type 1 diabetes (T1D) is not only increasing in incidence worldwide, but also in prevalence as treatment advances enable more children to live into adulthood [1–3]. Since the landmark findings put forth by the Diabetes Complications and Control Trial, T1D treatment and management centers about maintaining glycated hemoglobin (HbA1c) below 7% to prevent morbidity and early mortality [4,5]. To support individuals’ achievement of glycemic targets, consensus guidelines recommend a minimum of quarterly visits with a multidisciplinary clinical care team since the complexity of T1D requires ongoing adjustment to treatment and self-management approaches [6,7].

The transition between childhood and adulthood is a physiologically and behaviorally challenging stage during which individuals with T1D all around the world are least likely to meet target glycemic levels, despite being a crucial period for adopting healthy lifelong habits to prevent later adverse health outcomes [8–10]. Although maintaining contact with clinical care during this stage is associated with better glycemic management and fewer acute care visits, long gaps in care are especially common during this period and clinic attendance often declines after transfer to adult care [11–15].

To improve care engagement and glycemic management during emerging adulthood, consensus guidelines promote beginning an intentional transition process from early adolescence that includes gradual practical and psychological preparation to assume self-management tasks independently and navigate the adult clinical care setting and treatment encounters [6,16–20]. In developed settings, there is a maturing evidence base of behavior change and care transition strategies that may improve care engagement and glycemic management during this stage [21–23]. However, weaknesses in this evidence base include few strategies informed by theory, multiple stakeholder perspectives, or rigorously tested via randomized control trials [15,23]. Further, to-date there is no published information about formal care transition programs in lower resource settings, and even more broadly, there is a paucity of evidence about barriers to or strategies for behavior change among young people with T1D in these contexts.

Our formative qualitative work among providers, patients, and parents across a sample of private and public clinics in Delhi, India indicated differences in system, clinic, and patient-level factors relevant to the transition process and post-transfer outcomes as compared to the higher resource settings that dominate the existing evidence base on emerging adults with T1D [24,25]. Informants indicated heterogeneous management of transition processes in the clinical landscape across India (e.g., transfer age, counseling practices) but no formal protocols or programs in place. Providers across private and public settings estimated only about 10–50% of their T1D patient population followed up with them at least every three months as advised. Among the emerging adults we planned to target with the intervention, informants explained that a visit frequency of once every two to even three years was not uncommon due to geographic displacement, sub-optimal provider and patient rapport, and low self-management readiness and motivation due to various social and economic factors and priorities [24].

Health facilities in India also vary widely within and across public and private settings as regards provider training, treatment and prescribing practices, patient to provider ratios, patient socioeconomic status and out-of-pocket costs. Whereas a visit with an endocrinologist at a public facility could be as low as 0–10 Indian rupees and include a supply of basic insulin (e.g., regular and Neutral Protamine Hagedorn [NPH]), the cost of a consultation at a private facility could reach up to 2000 Indian rupees, exclusive of insulin. As such, because most health care costs are borne by the patient in India, patients of public facilities are more likely to be low or middle income, uninsured, have low literacy, and be unable to afford insulin, blood glucose test strips, or advanced diabetes self-management technology; in contrast, patients of private facilities are more likely to be insured or have a level of income that affords private clinic consultation fees, analog insulin, and routine use of continuous glucose monitoring systems and insulin pumps. Because pediatric endocrinology is not a widely prevalent specialty, adult physicians may treat children from T1D diagnosis and pediatric physicians may treat children from T1D diagnosis into adulthood. Gaps in knowledge therefore persist regarding how to adapt and implement care strategies in lower resource contexts such as India that were developed and evaluated in developed settings, as well as the effectiveness of such strategies in these contexts.

Additionally, detailed reports about the development processes that underpin interventions are extremely limited, which poses obstacles for intervention replication and improvement efforts [21,26]. More widespread publication of these reports and use of consensus-based reporting guidelines to structure them could not only promote learning within and across fields of study, but also promote scientific rigor in the intervention development process by promoting consideration of theory and empirical evidence before undertaking intervention design and implementation. Such transparent reports of programs in developing settings are notably absent despite the arguably greater importance of shared learning to expedite development of evidence-based interventions in the context of greater resource constraints and absolute burden of disease.

The objective of this article is to report the process and outcomes of developing a formal program to improve frequency of routine clinical care attendance and self-management among emerging adults with T1D in Delhi, India after transfer from pediatric to adult care. A systematically developed, theoretically and empirically sound program is foundational to our subsequent aims to rigorously test the program via a randomized controlled trial (CTRI/2020/10/028379), modify the program for additional and larger scale evaluations, and ultimately, inform clinical practice guidelines for managing T1D care transitions in India. Through standardized, detailed reporting of the intervention development process and resulting program, we also contribute to efforts to increase rapid development, testing, and sharing of theory, evidence, and stakeholder-informed transition of care interventions for emerging adults with T1D in India and other resource limited settings.

## Methods

The central intervention development team was mainly comprised of endocrinologists, diabetes educators, and researchers with training in epidemiology and behavior change who had context-specific expertise in pediatric and adult T1D and who were employed by a public research hospital in Delhi, India that was also an intervention implementation site. We followed the GUIDance for the rEporting of intervention Development framework to report each step of the intervention development process (S1 Appendix) [26]. The intervention is reported using the Template for Intervention Description and Replication (TIDieR) checklist (S2 Appendix) [27]. Both the formative research study that involved human participants referenced in this report as well as the future randomized controlled trial that will evaluate the program were granted ethical clearance (REF: IEC-82/01.02.2019, RP-27/2019) by the institutional ethics committee of the All India Institute of Medical Sciences, New Delhi.

A guiding principle of the intervention development process was to promote likelihood that the program could be integrated into existing clinical care practice and widely disseminated across more clinical settings in India if proven effective. As such, a priority was for intervention sessions to coincide with quarterly clinic visits–the minimum recommended frequency of clinical care visits according to consensus guidelines [6,7]. A related priority was for sessions to be delivered as much as possible by existing staff across clinics of different sizes, pay models, counseling practices, staff training backgrounds, and patient socioeconomic status. Because our formative work indicated clinician buy-in was a foundational step for widespread adoption of changes in clinical practice and eventual change in administrative policy, clinicians were asked for input at multiple points of the intervention development process, as described below [24].

In developing the **P**ediatric to **A**dult **T**ransition Care for the **H**ealth and **W**ellness of **A**dolescents with **Y**oung Diabetes in India program (PATHWAY), we used a “combination” approach by integrating three formal approaches to intervention development [28]. Starting with a “theory and evidence-based” approach, we identified a list of potential intervention components through an iterative literature review of published research evidence and theories both specific to improving clinical engagement and health outcomes among emerging adults with T1D in the transition between pediatric to adult care as well as general to emerging adults with youth-onset chronic conditions [21–23,29]. Subsequent steps were informed by both a “target population-centred” approach (i.e., attention to views and actions of those using or benefiting from the intervention) and “implementation-based” approach (i.e., attention to the intervention being used in the real world). A stakeholder mapping exercise identified key stakeholder groups involved in T1D care transitions, after which a formative research and analysis phase was undertaken over a 9-month period (May 2019 -March 2020) to refine important intervention targets and elicit suggestions about intervention components, which included in-depth interviews with 38 patients, parents, and providers across public and private clinical settings. Detail about the design and results of the in-depth interviews conducted with key stakeholders is included in a report of our formative research [24].

In December 2019, via a 3 hour in-person workshop with 40 providers from prospective public and private intervention sites, the central research team presented and elicited feedback on the idea of a formal transition program as well as potential intervention and program design options identified through literature review and preliminary findings from the formative research.

A series of bi-weekly one hour working sessions with the central research team were subsequently held over a one-year period to integrate evidence from the formative research, published literature, and clinician feedback, in order to first draft the structure of the intervention at a high level (i.e., intervention length, frequency and objectives of sessions, roles and responsibilities), and then to draft session content and material in detail.

To prioritize and select the constructs that the intervention would target at provider and patient level (i.e., interpersonal and psychosocial factors), we combined context-specific insights from the formative research together with evidence gathered through our literature review, as well as the expanded Social-Ecological Model of Adolescents and Young Adult Readiness for Transition (SMART), an empirically developed, stakeholder and social-ecological theory informed model of the social (e.g., economic status), interpersonal (e.g., provider relationship), and individual-level (e.g., motivation) factors that shape transition readiness and related health outcomes [6,16,19–22,30–34].

Once the high-level design of the intervention was established, the development of specific session activities and materials began with aggregating strategies and content from published health care transition and behavior change studies in emerging adults with T1D, or recommended by T1D or health care transition consensus guidelines to address the priority constructs [21,22]. These strategies and materials were then tailored to be relevant to the context of Delhi, India in which they were to be implemented based on insights from the central research team, formative research. The intervention structure, session activities, and materials were then presented in detail to pediatric and adult providers from prospective implementation sites to elicit feedback. Due to the need to hold the workshops virtually because of the ongoing COVID pandemic, two 1–2 hour online conference sessions were held with 43 pediatric and adult providers in October 2020.

## Results

### Findings from stakeholder engagement and influence on PATHWAY program design

Reinforcing findings from our qualitative formative interviews, clinician participants in the stakeholder workshop held in December 2019 expressed unanimous endorsement of a formal transition program [24]. Due to the reality of already time-strapped and insufficient staff at most clinics, participants further emphasized the infeasibility of full reliance on existing staff to take on the additional tasks involved in a transition program, as well as the infeasibility of a joint clinic that would involve time-consuming geographic displacement of providers. This feedback influenced both design and implementation through the decision to incorporate an ‘overlap phase’ into the program (i.e., time period during which the patient sees both adult and pediatric provider) as well as to hire additional staff to support program delivery.

At the follow-up workshops held in October 2020, participants were particularly concerned about successfully convincing patients to switch providers and thus emphasized the importance of incorporating repeated efforts from multiple transition program actors to persuade the patient about the rationale for transfer and manage any misunderstandings that might discourage them from seeking care. This feedback resulted in developing consistent messaging about the rationale for transfer and designing sessions so that this messaging was repeated by adult and pediatric physicians and diabetes educators throughout the program. Workshop participants also suggested that the control group be provided with slightly more support than just receiving notice of the deadline for transfer to adult care in order to motivate their participation and so that the control condition approximately mimicked the most supportive way transfer is currently managed in the existing clinical landscape. This feedback influenced the decision for the control group, and thus the PATHWAY transition program comparator condition, to represent a type of ‘bare bones’ or ‘minimalist’ one-session transition program.

As previously described, a comprehensive report of the results from the qualitative formative interviews has been published elsewhere [24]. The way stakeholder feedback obtained throughout the intervention development process underpinned specific transition program design elements, is further detailed in the subsequent section that describes the PATHWAY program.

### Description of the PATHWAY program design: Logic model, program structure and content

**Fig 1:**
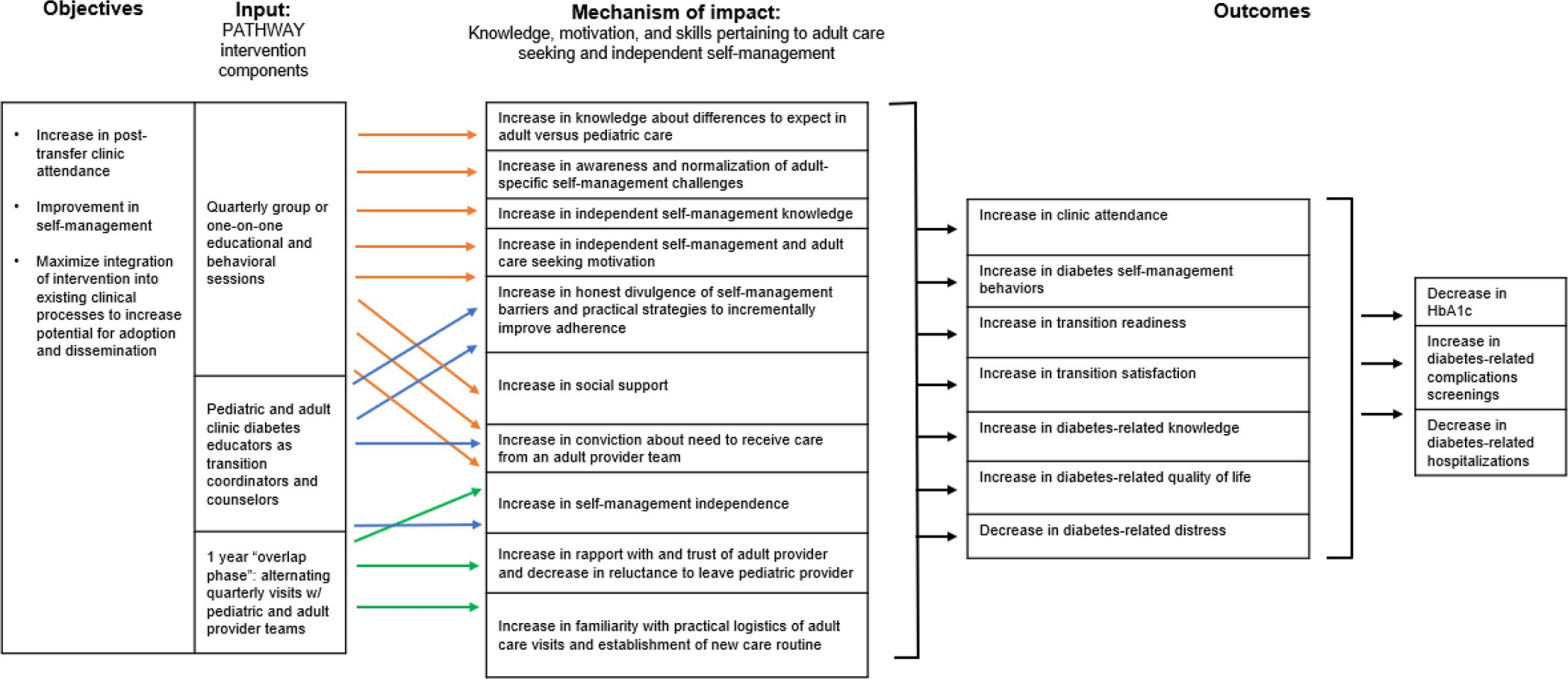
The PATHWAY intervention logic model.

The PATHWAY transition program that will be tested in a future randomized controlled trial is described below. The logic model in Fig 1 depicts how the program components achieve the intervention target outcomes and objectives [35–37]. The 15-month structured transition program has three primary defining features: diabetes educators as the central coordinators and counselors of the transition program, a one year “overlap period” during which time the emerging adult with T1D alternates between adult and pediatric provider team visits, and quarterly counseling sessions focused on targeting theoretically and empirically supported psychosocial factors associated with clinical care engagement and other positive outcomes during health care transitions among emerging adults with T1D [21–23].

A growing evidence base suggests post-transfer clinical care engagement and glycemic management are improved through structured transition interventions that include both a “transition clinic” to build rapport with the adult diabetes providers (i.e., emerging adults visit with both pediatric and adult providers at a jointly staffed clinic before permanent transfer) and “transition coordinators” who counsel emerging adults on practical and psychosocial factors that impact clinical visit attendance and self-management adherence in adulthood [22,23]. Our context-specific formative work and feedback from workshops with clinicians indicated that joint visits would be infeasible due to the common occurrence of patients switching health care institutions when transferring between pediatric and adult providers. Further, high patient-to-provider ratios challenge the coordination of pediatric and adult physician schedules even when both providers work within the same institution. Thus, in order to facilitate rapport-building and adult physician familiarity with the emerging adult’s diabetes, PATHWAY involves an “overlap period” during which time participants alternate quarterly clinic visits between pediatric and adult provider teams for a yearlong period before official and permanent transfer to the adult care team. These visits combine meetings with the physician and supplemental psychosocial counseling sessions–described below—by the diabetes educators at each site. This quarterly intervention session frequency throughout the 15-month transition program intervention aligns with the minimum recommended frequency of routine clinical care visits for individuals with T1D, consistent with our objective to develop an intervention that could be more readily built into routine care if proven beneficial. In addition to facilitating gradual rapport building with the adult physician and diabetes educator, this program format addresses the practical and psychosocial factors that influence clinical care engagement and increases convenience of the transition program for patients–many of whom travel long distances and may be deterred by the requirement to make additional trips to the clinic outside of those made as part of routine care.

Our formative research also highlighted several context-specific reasons to position diabetes educators at the center of the structured transition program as transition coordinator and counselor. One of the facilitators of care engagement and diabetes self-management most commonly cited in our formative research included trust that the adult provider not only understands the emerging adults’ diabetes treatment regimen, but also empathizes with and helps address the psychosocial factors that shape an emerging adult’s self-management difficulties [24]. Given the case loads of adult endocrinologists in Delhi, India, which make short encounters and case sharing among physicians common features of adult diabetes care visits, informants described the importance of enlisting providers outside of the adult endocrinologist to provide psychosocial support during the transition process to reduce likelihood that care engagement would hinge on establishing what was perceived as an often infeasible, idealistic relationship with the adult physician. Compared to other providers in diabetes care teams across the clinical context in which PATHWAY was designed to be implemented, diabetes educators were described as having the most skills and time to establish rapport with emerging adults and address psychosocial and practical barriers to care engagement and self-management issues during the turbulent period of early adulthood. Our design of PATHWAY to maximize delivery of the educational and behavioral intervention sessions by the diabetes educators employed at private and public pediatric and adult clinics also aligned with a key development objective to produce a program that could be implemented and sustained within the existing clinical landscape. However, given the multiple clinical responsibilities that site diabetes educators manage outside their delivery of PATHWAY, 4 additional diabetes educators (2 nurse educators and 2 dieticians experienced with T1D) were hired in order to coordinate program scheduling, oversee assessments requiring completion as part of the clinical trial, and deliver sessions when site-specific diabetes educators could not provide a session on a date and time that overlapped with participants’ quarterly visits.

Our review of published studies and expert consensus guidelines about care transitions and behavior change among adolescents and young adults with type 1 diabetes also underscored the importance of proactively managing transition-related psychosocial factors in order to improve care engagement and health outcomes after transfer: attachment to and reluctance to leave pediatric provider, lack of awareness of transition timeline, unfamiliarity with differences in adult provider approaches, expectations of patients, and visit procedures [21,23]. Additionally, the evidence base suggests promoting transition readiness, care engagement, and other outcomes by targeting additional factors that influence self-management and care seeking behaviors: self-management knowledge, problem-solving and goal-setting skills, motivation, self-efficacy, social support, emotional regulation, and conflict management [21–23].

**Fig 2:**
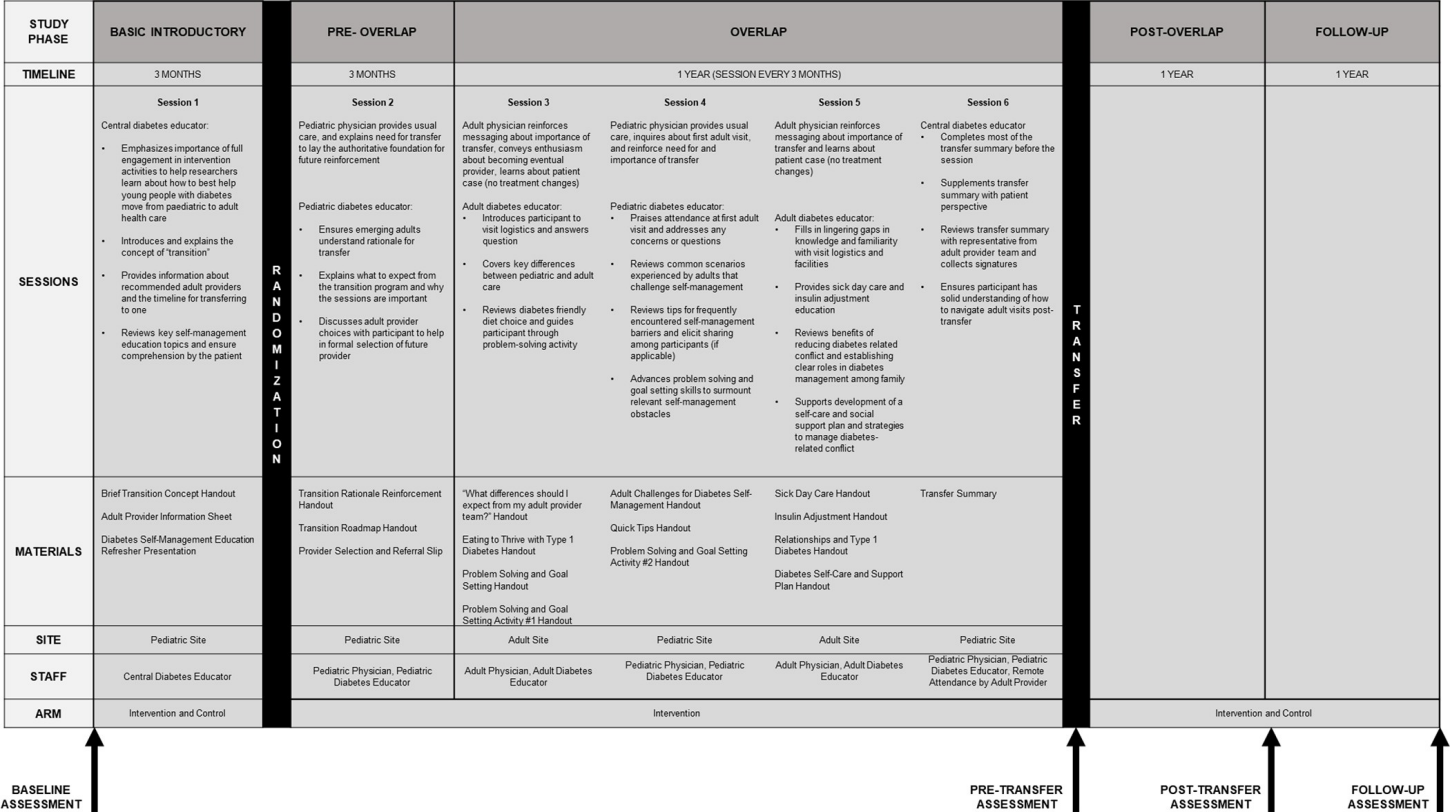
Detailed description of PATHWAY intervention program timeline, sessions, materials, sites, and staff.

Although our formative work reinforced the importance of all these factors to varying degrees, when designing the intervention sessions and their comprising educational and behavioral activities, we targeted the factors that were especially salient according to our context-specific investigations (Table 1). The materials used to facilitate session activities were also informed by our literature review and tailored for the context of intervention implementation based on our formative work that elicited perspectives from patients, parents, and clinicians, as well as the expertise of clinician, behavior change, and public health members of the central research team. A session-by-session description of intervention design and activities can be found in Fig 2 and corresponding materials used can be found in S3 Appendix.

**Table 1:** Factors targeted through design of PATHWAY intervention to promote post-transfer clinic attendance and other health outcomes.

Intervention targets	Intervention session activities
Inadequate knowledge, skills, and motivation to navigate self-management in adulthood	Conduct education, problem-solving, and goal-setting exercises specific to scenarios that commonly challenge the self-management of adults with type 1 diabetes: food choices; sick day care; insulin adjustments; reproductive health; relationship conflicts; unpredictable schedules; self-management in work, college, and social settings;
“Outsourcing” of diabetes management to caregivers sustained through adulthood	Establish a clear responsibility-delineation plan outlining the self-management tasks to be assumed by patient vs. family members, and set goals for gradual increase in self-management independence
Reticence to divulge adherence barriers to clinically recommended behaviors	Through group education and experience sharing, normalize adherence barriers and develop strategies for incremental progress in aligning self-management behaviors with clinical recommendations
Misinterpretations that transfer to new provider is fault of emerging (i.e., “bad patient”)	Introduce and reinforce the social, behavioral, and physiological reasons that transfer is in the best interest of the participant
Attachment to pediatric provider and no pre-transfer rapport with adult provider	Enlist pediatric provider to explicitly communicate support of transition, provide list of adult provider options, facilitate pre-transfer visits with adult provider team to build rapport, and develop summary of relevant medical and psychosocial information to build patient trust and adult provider familiarity with patient case
No expectations established in advance about transition timeline or differences in adult care visits or approach	Provide advanced awareness of transition timeline, prepare emerging adults for differences to expect between pediatric and adult care through pre-transfer education and visits with adult provider

We decided upon several main changes in transition program content and structure during our bi-weekly intervention development team meetings. First, findings from our formative research and first stakeholder engagement workshop led us to revise our plans to completely rely on the clinic employed diabetes educators to coordinate and deliver the program. Four diabetes educators were employed by the intervention development team to support session delivery during the randomized controlled trial so as to prevent against over-extending existing resources at participating sites, which could dilute fidelity in intervention delivery, lower the rigor with which the strategy was tested through the randomized controlled trial, and reduce provider buy-in for the program. Although this design decision will challenge immediate translation of the program into existing clinical practice if trial results indicate program effectiveness, information gathered from the planned RE-AIM evaluation (i.e., monitoring checklists and interviews with providers and patients) of the randomized controlled trial will generate insights about the staff time and monetary resources that may be required by practices to implement the strategy as well as generating suggestions about the components of the program that may be particularly important for driving target outcomes. These insights could inform a strategy to pare-down the program design to increase feasibility and uptake into existing settings, which could then be assessed for effectiveness through a future evaluation. Second, after the second stakeholder engagement workshop, we made adjustments to deliver a session (Session 1, Fig 2) to all study participants before randomization (i.e., both control and intervention arms) in order to compare the 15-month transition program to a minimalist, one-session transition program, Third, the intervention sessions were initially conceived to be group sessions to reduce staff implementation burden, promote normalization of adherence barriers, and facilitate instrumental and non-instrumental peer support that have been demonstrated to enhance self-management during this developmental stage [21,23]. We introduced flexibility to deliver these sessions in either group or individual format due to challenges aligning patient schedules and because we anticipated more gradual trial recruitment due to COVID-19.

## Discussion

This report provides an example of systematic, evidence-based, and stakeholder-informed development of a context-specific care transition program to promote care engagement and self-management in emerging adults with T1D in Delhi, India (“PATHWAY”), which we plan to rigorously test in a randomized controlled trial in future steps. Strengths include integrating the evidence base from high resource settings together with context-specific stakeholder perspectives from our formative work in order to identify the intervention targets and strategies that define the program. Such an approach may increase the potential that the program promotes intended outcomes among emerging adults with T1D in Delhi, India. Further, our use of GUIDED and TiDIER reporting frameworks (S1 Appendix and S2 Appendix) to transparently report the intervention development process. Employment of these reporting frameworks facilitates comprehension of the program design and may inform development of theoretical and evidence-based care transition strategies for emerging adults with T1D in other resource limited settings, which are currently lacking despite growing incidence and prevalence of this disease in these settings.

Limitations and persisting uncertainties include that the care transition evidence base and our formative research that informed the PATHWAY program were undertaken before COVID-19, which introduce the possibility that unexplored intervention targets and strategies could be relevant to our target population and setting, but are not incorporated into program design. Although we will not systematically collect information to address this point of uncertainty before the program is tested through a clinical trial, a planned mixed methods Reach, Effectiveness, Adoption, Implementation, and Maintenance (RE-AIM) evaluation of the trial will help to identify these factors and enable future refinement of the program. We purposively designed the program to rely on the diabetes educators employed by adult and pediatric clinics to deliver as much of the intervention as possible in order to minimize program expense and maximize potential that the program would be more widely adopted and tested if proven effective through our initial randomized controlled trial evaluation. However, this choice also introduces risk of low and variable fidelity across implementation sites. To promote fidelity and prevent drift, we designed scripted counseling materials to facilitate consistent implementation across sites and we will also monitor a random subset of intervention sessions throughout implementation as part of the trial. The aforementioned RE-AIM evaluation will enable us to understand differences in fidelity across sites and time, identify factors that potentially underly these differences, as well as explore the influence of these factors on the trial outcomes observed.

Detailed reporting of interventions and their underlying processes are limited, especially in the context of resource limited settings and T1D care transitions. Our systematic development and reporting of the PATHWAY program is an important foundational step towards identifying strategies that may benefit the self-management of emerging adults with T1D in India and similarly resourced settings.

## Supporting information

S1 Appendix GUIDED checklist. (DOCX)

S2 Appendix TIDier table. (DOCX)

S3 Appendix PATHWAY transition program session materials. (PDF)
