# Supplementary material for: Development and design of the first structured clinic-based program in lower resource settings to transition emerging adults with type 1 diabetes from pediatric to adult care
Source: PLOS Glob Public Health. 2022 Aug 3;2(8):e0000665. doi: 10.1371/journal.pgph.0000665 (PMC10021365; doi:10.1371/journal.pgph.0000665)
Supplement: S2 Appendix — (DOCX) [file pgph.0000665.s002.docx]

**S2 Appendix – TIDieR checklist**

| **Item number** | **Item** |  |
| --- | --- | --- |
|  | **BRIEF NAME** |  |
| **1.** | Provide the name or a phrase that describes the intervention. | **P**ediatric to **A**dult **T**ransition Care for the **H**ealth and **W**ellness of **A**dolescents with **Y**oung Diabetes in India program (PATHWAY) |
|  | **WHY** |  |
| **2.** | Describe any rationale, theory, or goal of the elements essential to the intervention. | The PATHWAY intervention seeks to promote clinic attendance and self-management in emerging adults with type 1 diabetes (T1D) after transfer to adult care using an educational and behavioral program format and content that can be integrated into existing clinical practice. |
|  | **WHAT** |  |
| **3.** | Materials: Describe any physical or informational materials used in the intervention, including those provided to participants or used in intervention delivery or in training of intervention providers. Provide information on where the materials can be accessed (e.g. online appendix, URL). | Materials used as part of intervention session delivery – Appendix C |
| **4.** | Procedures: Describe each of the procedures, activities, and/or processes used in the intervention, including any enabling or support activities. | *First Session – Before Randomization (Intervention and Control Arm; Pediatric Site; Bridge Diabetes Educator)*  This session, attended by all study participants pre-randomization into control and intervention arms, represents a one session transition program with minimal education components that are included in the more comprehensive procedures used to manage transitions across the current clinical landscape of study implementation. This minimal transition intervention consists of making emerging adults aware of a formal policy requiring transfer after 18 months, providing a list of available adult providers in Delhi, and reviewing self-management education topics covered in pediatric care.  *Second Session – Before 1 year Overlap Phase (Intervention Arm; Pediatric Site; Pediatric Physician and Diabetes Educator)*  The paediatric physician provides usual care to the participant and introduces the need and rationale for transfer to adult care, which leverages rapport with the patient to lay an authoritative foundation for convincing the patient about the importance of transfer, which the diabetes educator will then reinforce and also work through any concerns and misunderstandings the participant may have. The diabetes educator will subsequently ensure in-depth comprehension by the participant by walking through the “Transition Rationale Reinforcement” handout with the participant. Subsequently, the diabetes educator will make the participant aware of what to expect from the transition program (“Transition Roadmap” handout ) which will increase the participant’s knowledge of the transition process and help them understand the value of fully engaging with the process. The diabetes educator will additionally provide the opportunity to discuss adult provider choices before formal selection adult provider by completing and signing the provider selection and referral slip together.  *Third Session - During 1 year Overlap Phase (Intervention Arm; Adult Site; Adult Physician and Diabetes Educator)*  This is the participant’s first visit at the adult provider site. This visit addresses apprehensions which may arise due to lack of familiarity as regards the visit procedures, clinic facilities, and providers. The adult diabetes educator plays a key role in setting the tone for the participant’s experience in adult care going forward by increasing their practical knowledge and trust through 1) conveying eagerness of being able to support participant in diabetes management as an adult 2) touring the participant around the physical environment, walking the participant through the logistics of a visit in the adult facility, and 3) helping the participant anticipate differences between adult and paediatric care (“What differences should I expect from my adult provider team” handout). During this introduction the adult diabetes educator will probe for questions or concerns and introduce the participant to the adult physician. In this encounter, the adult physician will reinforce the importance of transfer for the good of the participant, consistent with the messaging provided by the paediatric physician and diabetes educator at the previous visit. Additional diabetes educator-delivered education includes teaching/reviewing diabetes friendly diet choices (“Eating to Thrive with Type 1 Diabetes” handout), introducing the participant to the importance of problem solving for successful independent self-management of diabetes (“Problem Solving and Goal Setting” handout), and completing a problem solving exercise to help the participant gain practice applying these new problem solving skills to be more successful at following a diabetes friendly diet (Problem Solving and Goal Setting Activity #1” handout). The adult physician will praise the participant for their engagement in the transition program and reinforce the messaging from the other providers about the importance of transfer. The adult physician will also review the patient’s diabetes treatment regimen to increase familiarity with the participant’s diabetes and get to know the participant to build rapport and comfort. However, the participant will not receive any treatment from the adult physician until the point of transfer.  *Fourth Session - During 1 year Overlap Phase (Intervention Arm; Pediatric Site; Pediatric Physician and Diabetes Educator)*  In addition to providing usual care, the paediatric physician will inquire about the first adult visit, congratulate the participant on achieving that milestone, and again reinforce the need for and importance of transfer to help motivate the participant to continue on in the transition process and be increasingly convinced of the importance of adult care. The remainder of the session is led by the pediatric diabetes educator, who will also praise the participant for a successful visit, and help resolve any concerns, questions or misunderstandings the participant may have. Next, the paediatric diabetes educator will review scenarios that adults experience that can make it hard for them to prioritize their diabetes self-management and be in good control (“Adult Challenges for Diabetes Self-Management” handout) to increase participant awareness and anticipation of common challenges, and help normalize the challenges. To increase participant knowledge of strategies to address common self-management challenges that adults face, the pediatric diabetes educator will review ideas for frequently encountered barriers to diabetes self-management in adult life (“Quick Tips for Common Adult Diabetes” handout). To improve participant ability to successfully problem solve and surmount challenges for independent self-management, the pediatric diabetes educator will then help the participant practice using one of the scenarios just reviewed that is relevant and challenging to the participant (“Problem Solving and Goal Setting Activity #2” handout). The suggested tips can be used to help the participant design their goal to surmount the challenge they select for the problem-solving activity  *Fifth Session - During 1 year Overlap Phase (Intervention Arm; Adult Site; Adult Physician and Diabetes Educator)*  This is the second and last visit to the adult site before the subsequent “transfer summit” that marks the participant’s formal and permanent transfer to adult care. This session is therefore an important opportunity to increase participant comfort with the adult site and provider team and fortify skills and readiness for independent diabetes self-management. The adult physician will encourage the participant for their progress in the transition program, and again reinforce the messaging from the other providers about the importance of transfer. As in the first encounter with the adult physician, the adult physician will build more familiarity with the participant and their diabetes. The adult diabetes educator will fill in lingering gaps in participant knowledge and comfort with visit logistics and clinic facilities, as well as reinforce messaging from the adult physician to motivate participant conviction about transfer importance. The adult diabetes educator will then refresh patient knowledge of sick day care (‘Sick Day Care” handout) and insulin adjustment and addresses and questions or misunderstandings (‘Insulin adjustment” handout). After reviewing the importance of establishing clear roles in diabetes management among family/close friends and reducing conflict about diabetes (“Relationships and Type 1 Diabetes” handout), after which the diabetes educator helps the participant create a practical adult plan for diabetes care responsibilities and strategize ways to manage diabetes related conflict (“Diabetes Self-Care and Support Plan” handout)  *Sixth Session - During 1 year Overlap Phase (Intervention Arm; Pediatric Site; Representatives from Pediatric and Adult Provider Teams)*  This session formally marks the participant’s last visit to the pediatric provider site and permanent transfer from paediatric to adult care. This visit ensures the participant has a solid practical understanding about how to receive care at the adult facility and also ensures transfer of key information about the participant to the adult providers so they are prepared to support the physiological, psychological and behavioural needs of the participant and help them thrive as an adult with diabetes. The central diabetes educator, after consulting with the paediatric provider, completes the transfer summary and supplements it with information from the participant perspective (i.e., in person at the visit, or previously over the phone) in order to enhance participant confidence that the adult provider is knowledgeable of their diabetes as well as their greatest concerns and challenges with diabetes self-management. Representatives of the adult and paediatric provider team are assembled either in-person or remotely (i.e., conference call). Ideally, the participant, the paediatric physician and diabetes educator, and adult physician and diabetes educator are assembled in person. However, it may be more likely that the adult physician and/or diabetes educator join remotely. Providers ensure the participant knows how to navigate scheduling and attending adult care visits independently and make the participant aware of what to expect after the transfer. Attendees sign the transfer summary to symbolize the permanence of the transfer and convey a respectful shift of responsibility from paediatric to adult provider. |
|  | **WHO PROVIDED** |  |
| **5.** | For each category of intervention provider (e.g. psychologist, nursing assistant), describe their expertise, background and any specific training given. | For each type of program session, site-specific diabetes educators (i.e., diabetes educators employed at paediatric and adult health care clinics) received a detailed explanation of the purpose and content of each session, as well as demonstrations using session specific scripts. Site-specific physicians and diabetes educators were provided a copy of the session scripts, participant-facing materials, and session checklists in a booklet. Diabetes educators delivered each type of session while being observed by the central study staff (using session-specific fidelity checklists) and were given personalized feedback on session delivery. The four bridge diabetes educators (employed by the central research team to deliver intervention sessions in the case that site-specific diabetes educators were unable to at the date and time of a participant’s visit) include two staff nurses and two masters level dieticians who also participated in the trainings. |
|  | **HOW** |  |
| **6.** | Describe the modes of delivery (e.g. face-to-face or by some other mechanism, such as internet or telephone) of the intervention and whether it was provided individually or in a group. | Face-to-face, group or individual |
|  | **WHERE** |  |
| **7.** | Describe the type(s) of location(s) where the intervention occurred, including any necessary infrastructure or relevant features. | Public and private diabetes clinics that treat pediatric and adult patients with type 1 diabetes in Delhi, India |
|  | **WHEN and HOW MUCH** |  |
| **8.** | Describe the number of times the intervention was delivered and over what period of time including the number of sessions, their schedule, and their duration, intensity or dose. | Quarterly ~30 minute educational, behavioral, and rapport-building sessions over a 6-month period. See Figure 2 for more detail. |
|  | **TAILORING** |  |
| **9.** | If the intervention was planned to be personalised, titrated or adapted, then describe what, why, when, and how. | NA |
|  | **MODIFICATIONS** |  |
| **10.^ǂ^** | If the intervention was modified during the course of the study, describe the changes (what, why, when, and how). | NA (intervention in initial stages of implementation) |
|  | **HOW WELL** |  |
| **11.** | Planned: If intervention adherence or fidelity was assessed, describe how and by whom, and if any strategies were used to maintain or improve fidelity, describe them. | Fidelity of intervention session delivery by each pediatric and adult clinical site delivering the intervention will be videorecorded and monitored via session checklists that evaluate delivery of the first session of each of the 6 sessions delivered as part of the study (Session 1-6). Personalized feedback will be provided to prevent drift in fidelity. Monitoring of video recordings of a random subset of intervention sessions at each site 6-12 months into implementation will enable longitudinal evaluation of fidelity over the implementation period. The qualitative component of the RE-AIM evaluation will provide insight into reasons for fidelity maintenance or drift over time. |
| **12.** | Actual: If intervention adherence or fidelity was assessed, describe the extent to which the intervention was delivered as planned. | NA (intervention in initial stages of implementation) |
