# Supplementary material for: Development and design of the first structured clinic-based program in lower resource settings to transition emerging adults with type 1 diabetes from pediatric to adult care
Source: PLOS Glob Public Health. 2022 Aug 3;2(8):e0000665. doi: 10.1371/journal.pgph.0000665 (PMC10021365; doi:10.1371/journal.pgph.0000665)
Supplement: S3 Appendix — (PDF) [file pgph.0000665.s003.pdf]

Appendix C

Basic Introductory Phase (Session 1)– Brief Transition Concept Handout

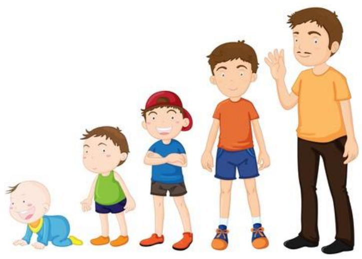

What is Transition??

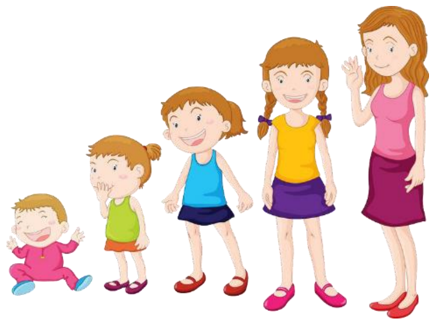

You celebrate your birthday every year. Every year you grow elder.
Once you were a child and now you are nearing adulthood. Once you were attending school and now you are already in high class (college).

Life is all about growing and attaining new milestones!!

SO, WHAT IS MEANT BY TRANSITION?

Transition means change in healthcare. It is a planned and purposeful transfer process through which adolescents and young adults move from child centric healthcare to adult centric healthcare.

YOU ARE BEING TRANSFERRED BECAUSE ...

You are an adult now!! In India, when a person crosses 18 years of age, he/she can no longer be seen by a paediatrician and therefore, has to seek care from an adult healthcare provider.

WHAT IS THE NEED OF THIS TRANSITION??

You are growing; your body is growing and changing. Your health issues are different now. An adult healthcare provider is better equipped to handle all your concerns and issues.

ANXIOUS?? DON'T BE!...

We are here to help you settle into the new healthcare clinic in which you will receive care as an adult. Please be honest with us about any concerns or questions you may have – we are here to help!

Basic Introductory Phase (Session 1) – Paediatric Self-Management Education Refresher

Introduce the reason for the refresher presentation: *Having the self-care knowledge and skills to manage your diabetes is a big part of feeling comfortable in adult care and being ready to take care of your diabetes independently. We will refresh our knowledge of some of these key diabetes self-care topics today, and share our challenges and tips for tackling these areas.*

The content of each module is explained in detail below:

Slide #3

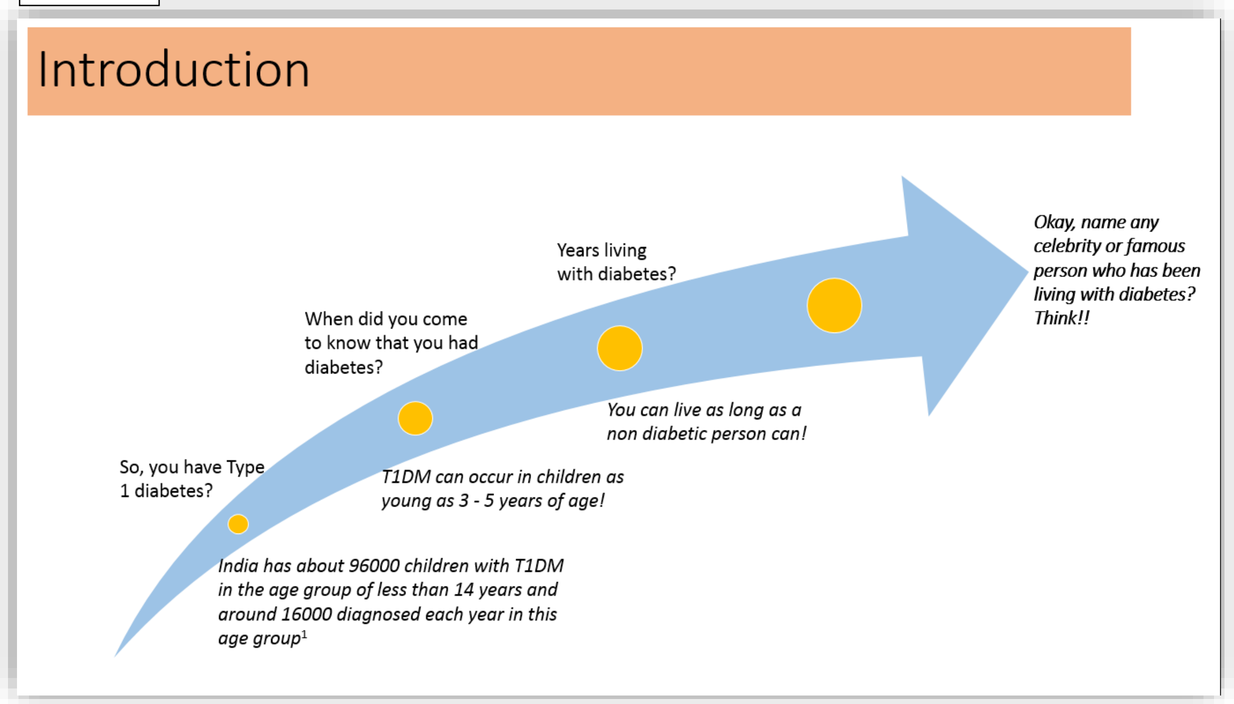

Begin with breaking the ice with the participant. This slide aims at establishing a rapport with the participant to enable the session delivery be an interactive one. In addition to the refresher presentation, a diabetes education booklet will be given to the DE that contains elaborate details on each of the modules mentioned above. This booklet is known as "Diabetes in children and young adults".

Help box (Slide #3)

	Okay, name any celebrity or famous person who has been living with diabetes?<br><br>***Answer: Sonam Kapoor (Actress), Kamal Haasan (Actor), Fawad Khan (Actor), Nick Jonas (husband of Priyanka Chopra), Wasim Akram (Cricketer)***	

For the slides on quiz time (placed before each module), they contain basic simple questions which aim at initiating a thought process in the participant’s mind related to that particular module. The quiz time is not to be judged or marked. It serves us a simple opening to the next module.

Slide #5

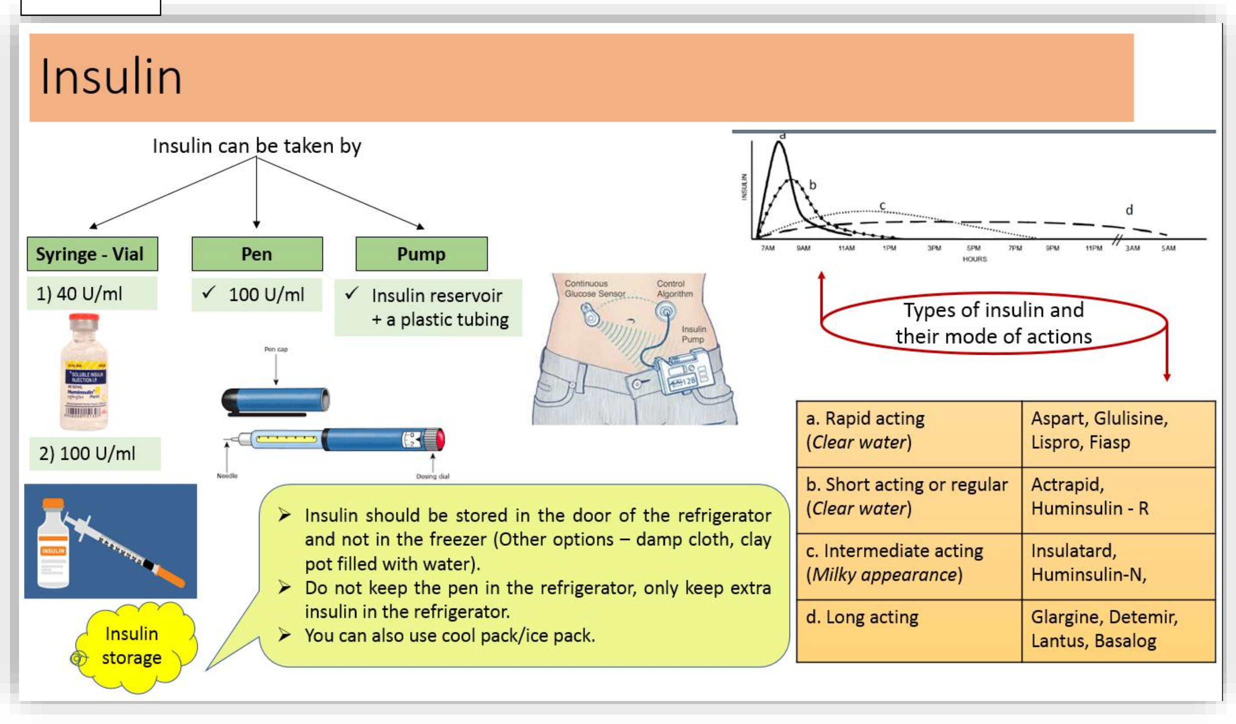

Begin with the need for insulin in Type 1 diabetes patients. The diabetes educator should thoroughly go through Chapter 1 and 2 in the “Diabetes in children and young adults” booklet, before explaining this slide. As previously stated, Type 1 diabetes is insulin dependent diabetes. Therefore, this slide should be explained bit by bit and no point should be left uncovered. Those who have diabetes from childhood (insulin dependent or type 1 diabetes) should necessarily take insulin injection every day throughout life.

3.1.4 Insulin:

There are two types of insulin – one which is short acting and provides coverage for meals (Short acting insulin e.g. Regular insulin or Rapid acting insulin analog like Aspart insulin, Lispro insulin, Glulisine insulin, Fiasp insulin) and the other which acts for longer time and provides basal glucose coverage (Intermediate acting insulin e.g. NPH (Neutral Protamine Hagedorn) insulin or long acting insulin analog like Glargine, Detemir, Degludec). Regular insulin is the one which is also made in our pancreas. It looks clear like water and is taken 3-4 times a day before major meals and occasionally with smaller snacks. Long-acting insulin

NPH is usually taken twice a day. It has a cloudy or milky appearance. It is made in the laboratory by adding some chemicals to the natural insulin to prolong its action. Some newer insulins are rapid acting insulin like Lispro insulin, Aspart insulin and Glulisine insulin. They can be taken just before meals (5-15 minutes before meal), instead of regular insulin (30 minutes before meal). Rapid acting insulin starts acting faster than regular insulin and remains active for a shorter duration. Your doctor will advise you as to when it should be taken and which patient can take it. Similarly, glargine or detemir can be used as long-acting insulin (Table 3.2)

Table 3.2: Types of insulin

Insulin as shown in the Slide #5, can be taken in three ways:

i. Syringe-vial
ii. Insulin pen

	**Rapid acting insulin** *(e.g. Aspart/ Lispro/ Glulisine)*	**Short acting** *(e.g. regular insulin)*	**Intermediate acting insulin** *(e.g. NPH)*	**Long-acting insulin** *(e.g. glargine/ detemir)*
Onset	15 minutes	30 minutes	2.5-3 hours	2-3 hours
Peak	1–1½ hours	2-3 hours	6-8 hours	--
Duration	3-4 hours	6-8 hours	12-16 hours	18-24 hours
Injection to be Taken	15 minutes before meal	30 minutes before meal	Morning and evening as per doctor’s prescription	As per doctor’s instructions
Expected action	Glucose control after food	Glucose control after food	Glucose control before food	Glucose control before food

iii. Insulin pump

Syringe-vial: Insulin is measured in terms of units. In India, we get insulin vial in two strengths - 40 unit per ml and 100 unit/ml. For 40 U/ml insulin, always use a 40-unit syringe. Similarly, for 100 U/ml insulin, use only 100-unit syringe. Additionally, a cartridge is always 100 U/ml. In case it ever becomes necessary to use a cartridge with a syringe, use it with a 100-unit syringe. Please take care always to match same unit insulin vial with same unit syringe.

Figure 4: Action profile of various insulins

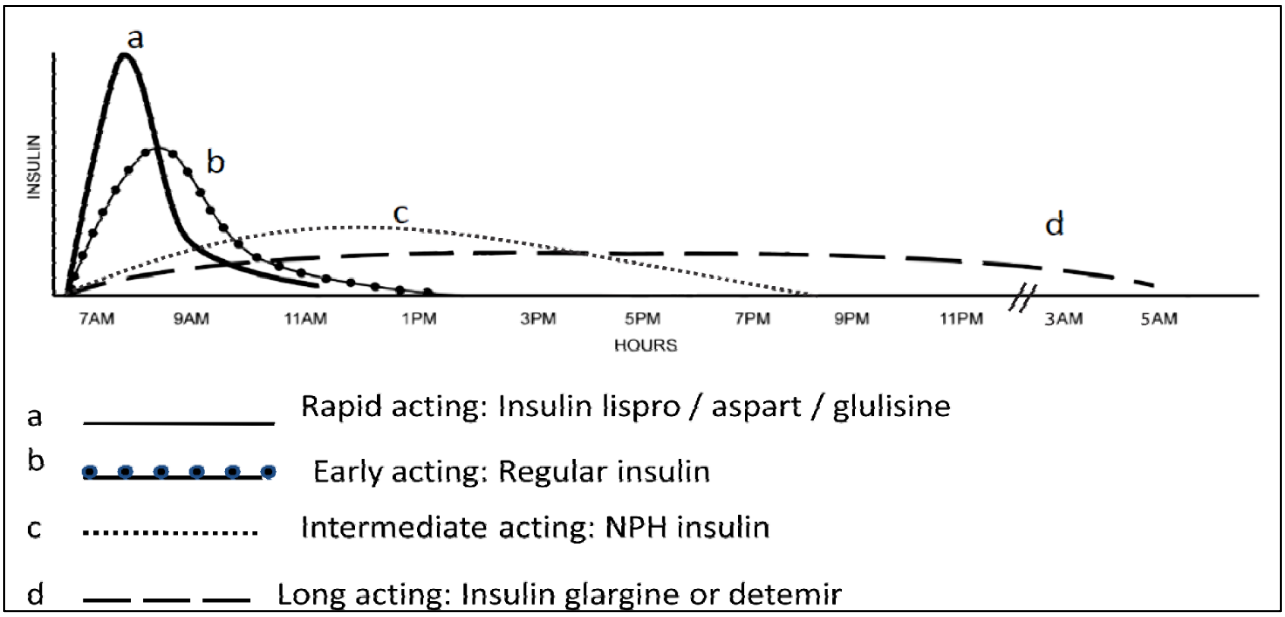

Insulin pen: Insulin injections can be taken by a pen instead of a syringe. This pen resembles a big fountain pen. Many people prefer the pen to the syringes even though they are costlier, as they are easier to use and carry to school/work. Remember, the insulin in a cartridge which fits into the pen is of 100 U/ml type; therefore, do not use it with a 40 U syringe (in case the pen is not working and you need to use the insulin in cartridge).

Insulin pump: This is a relatively new method to take insulin. A pump is like a small mobile phone in which a bottle of regular or rapid insulin is placed. A thin plastic tube carries the insulin from the bottle to the body just like the ordinary injection which you take. The only difference is that the tube remains in the injection site, under the skin, and is changed once in three days. The pump can be programmed so that it releases a greater dose of insulin before meal time (bolus dose) and small continuous doses when it is not meal time (a continuous or “basal” rate). This continuous dose takes the place of NPH or glargine insulin which are the basal insulin taken by pen or syringe. The basal rate can be pre-set at different rates, like at a lower rate for a few hours during midnight (to avoid hypoglycaemia) or at a higher rate to avoid the early morning rise in blood sugar experienced by many people. You can always take a bolus during snack time or meals, without having to use the needle again and again. You can keep better glucose control by using the pump. But for taking full advantage of the pump, you should test your blood glucose 4-7 times a day, and adjust your insulin dose according to the intended meal intake. At the moment, the cost of the pump and tube are a bit high. Please contact our team if you want to know more about this.

3.1.5 Method of drawing insulin:

1.Wipe the surface of the bottle with spirit and cotton. Wait for 2 minutes for the spirit to dry and disinfect the cap. Pierce the needle into the bottle and push in air (as much as at the units of insulin you will take).

2. Do not remove the needle from the bottle. Invert the bottle upside down and draw the plunger of the syringe downwards. Insulin will enter into the syringe. If there are air bubbles, tap the syringe while holding it upright and expel the air.

3. Now the syringe is ready for injection.

In case of mixed injections (regular and NPH combined)

1. Wipe the top of both regular as well as milky white NPH bottles with spirit and cotton.

2. Turn the bottle of NPH upside down and roll the bottle in the palm of your hand to mix the insulin nicely.

3. Draw the plunger down, to fill the syringe with air. Pierce the needle into the NPH bottle and inject air into the bottle. Remove the needle from the bottle. Similarly, fill air into the bottle of regular insulin. Let the needle remain in the bottle.

4. Invert the regular insulin bottle. Draw the desired units of insulin into the syringe, and remove the air bubbles.

5. Now insert the needle in the milky white bottle (NPH).

6. Allow the plunger to slowly come up to the level of the total mixed dosage. Remove the needle from the bottle.

Now there is appropriate mixture of regular and NPH insulin and this mixture is ready for use.

3.1.6 Method of injecting insulin:

Insulin may be injected in the arm, thigh, abdomen or buttocks, as shown in Figure 5. Insulin should be injected in the abdomen about two fingers away from umbilicus. While injecting into the upper arm, place the needle into the triceps area at the back of the arm, about half way between the elbow and the shoulder. In case of thigh, inject into the upper and outer area of the thigh. Avoid the inner thigh due to denser network of blood vessels in that area. Before injecting the insulin, wipe the skin with cotton and spirit and allow it to dry. Never wipe the needle with spirit. Hold the syringe like a pencil and pierce the needle directly into the skin. The needle can be pierced at right angle (perpendicularly) in adults, especially those with

adequate fat mass in the abdominal subcutaneous tissue. However, for children or lean adults who have little fat mass, it is better to inject at an angle of 45-60 degrees to avoid intramuscular injection. Slowly bring the plunger down. Press the spot where the injection has been given with cotton and spirit. Slowly remove the needle after making a count of 10. If you are using a pen, then remove the needle from the skin only ten seconds after delivering all the insulin, so that the medicine does not spill out. Sometimes a little drop of blood appears; do not worry, just press the spot with your finger for a minute. The correct method to give insulin is shown in Figure 6.

The site of injection should be rotated. Every new injection should be at a distance of 1 inch from the previous one. In this way if you have finished with one side of the arm and then the thigh, then you can start with the other side. Drawing a circle around the injection site with a ball point pen will help you choose a site 1" away for the next injection. If possible, do not take regular or rapid insulin in an exercising limb. Thus, you may prefer the abdomen for regular or rapid insulin and the thighs/ buttocks/arms for NPH or glargine insulin.

Figure 5: Site for insulin injection

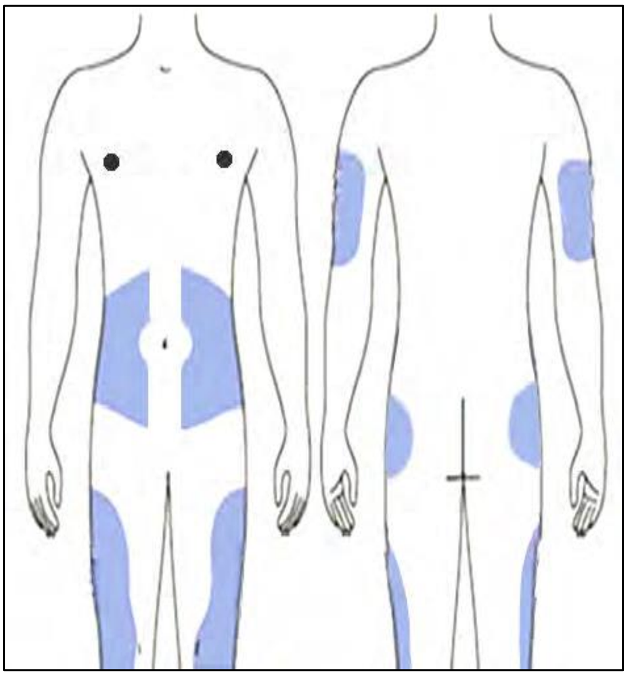

Figure 6: Insulin injecting technique

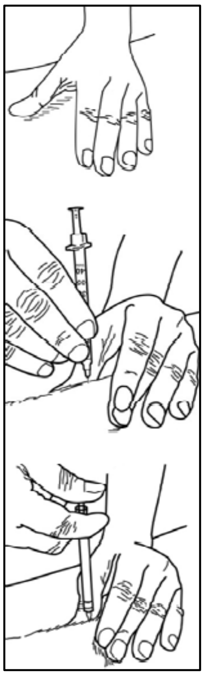

3.1.7 Storage of insulin:

Insulin should be stored in the door of the refrigerator. It should never be stored in the freezer. If refrigerator is not available, then wrap the insulin in a damp cloth and keep it in a cool shady place away from the sun. It can also be kept in a clay pot filled with water. Those who are using a pen should remember not to keep the pen in the refrigerator. Only extra

insulin cartridges should be kept in the refrigerator. The pen with the cartridge in current use can be kept in the room during winter months (between October- February). During summer months these may be kept in a cool pack. To know more about a cool pack, please ask anyone from the diabetes team.

Slide #7

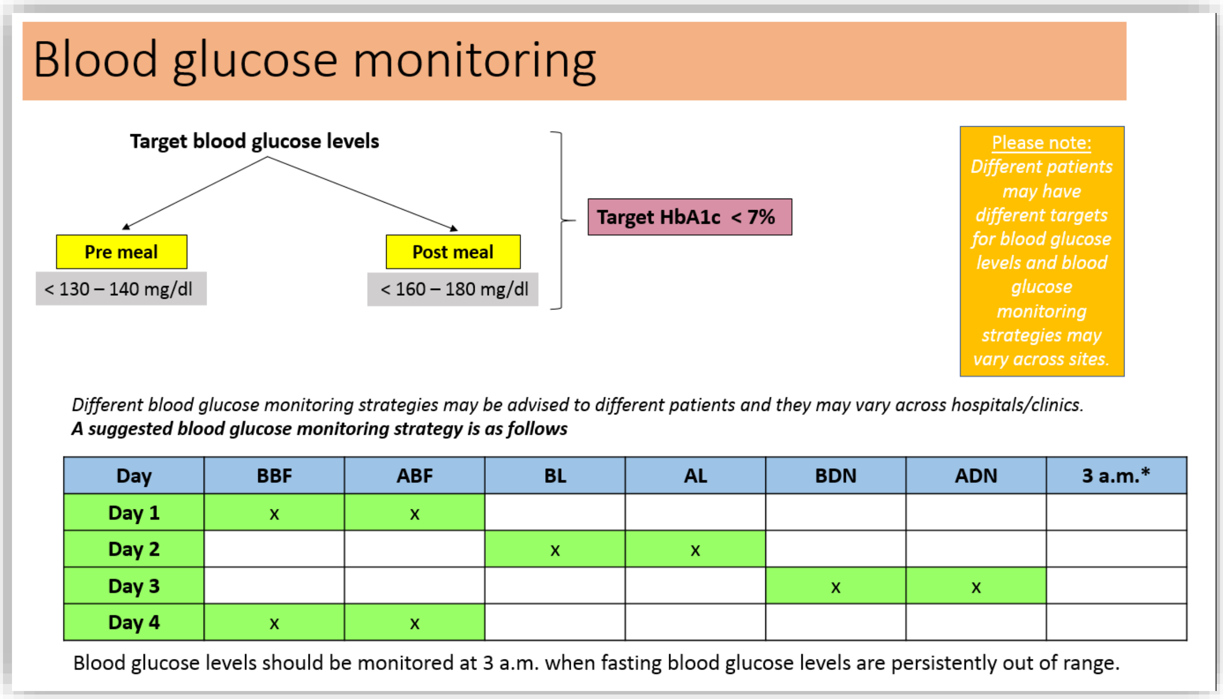

In slide number 7, you will mainly discuss about blood glucose monitoring. Begin with asking quiz questions about their routine blood glucose testing, their knowledge about hypoglycaemia and ideal blood glucose levels. Refer to Chapter 5 in the "Diabetes in children and young adults" booklet.

3.1.8 Blood glucose monitoring:

It is important to keep blood glucose under control to avoid complications of diabetes. Some tests can be done at home by the T1DM patient themselves and some can be done only in the laboratory. Measuring blood glucose at home is very necessary for good control of diabetes, because you can take immediate remedial action if blood sugar is too high or low.

For individuals who are young, have no comorbidities or diabetes- related complications and blood glucose control can be achieved without causing undue hypoglycaemia, fasting blood glucose (FBG) of $\leq$ 120 mg/dl (6.7 mmol/L) and 2-h postprandial blood glucose (PPBG) of $\leq$ 160 mg/dl (8.9 mmol/L) should be targeted.

For older individuals or those with comorbidities and diabetes-related complications for whom the risk of hypoglycaemia is higher, blood glucose targets should be relaxed to FBG ≤ 140 mg/dl (7.8 mmol/L) and PPBG ≤ 180 mg/dl (10 mmol/L). The upper limit for PPBG can be further relaxed to 220 mg/dl (12.2 mmol/L) if the efforts to achieve target value ≤ 180 mg/dl (10 mmol/L) are met with increased frequency of hypoglycaemia.

Different patients may have different targets for blood glucose levels and blood glucose monitoring strategies may vary across patients and across sites. A suggested blood glucose monitoring strategy is as shown in the Table 3.3 below:

Table 3.3: Blood glucose monitoring strategy

**Day**	**BBF**	**ABF**	**BL**	**AL**	**BDN**	**ADN**	**3 a.m. ***
**Day 1**	**X**	**X**					
**Day 2**			**X**	**X**			
**Day 3**					**X**	**X**	
**Day 4**	**X**	**X**					

*Blood glucose levels should be monitored at 3 a.m. when fasting blood glucose levels are persistently out of range. BBF – Before breakfast, ABF – After breakfast, BL – Before lunch, AL – After lunch, BDN – Before dinner, ADN – After dinner

3.1.9 Haemoglobin A1c

Haemoglobin A1c (HbA1c) is a test done in the hospital. It is an indication of the average blood sugar maintained in the last three months. Ideally, the normal value of HbA1c in a person without diabetes is 4.3 to 5.6%. However, in case of T1DM patients, care and effort should be taken to maintain HbA1c below 7% (Note: Different guidelines may apply to some situations; you should be individually guided by your diabetes care team).

Slide #9

Hypoglycaemia management

Hypoglycaemia is defined as blood glucose levels under 70mg/dl

Causes	Symptoms	Management
Inadequate meal or delayed meals –<br>• Missing meal<br>• Decreased carbohydrate content<br>• Delay in taking meal after having taken short acting insulin	• Trembling, weakness, irritability, hunger, sweating, anxiety	• Check your blood glucose immediately.
Excess insulin –<br>• Syringe vial mismatch (Example: using 100 IU/ml vial and 40 IU/ml syringe)	• Decrease in concentration during work	• Instant sources of energy – glucose powder, sugar, candies.
Exercise –<br>• Site of injection – e.g. rapid insulin in exercising limb	• Headache, double vision or darkness in front of the eyes	• Take rest – once the blood glucose level normalises, follow up with a carbohydrate snack.
Others –<br>• Vomiting<br>• Loose stools	• Inability to walk steadily	• Severe cases – glucagon injection
	• Anger	• Unconscious state – glucose powder paste should be applied on the oral mucosa
	• Unconsciousness or fits	• Friends/family should also be educated on hypoglycaemia management
		• Look for cause of hypoglycaemia to prevent future episode

The slide number 9, discusses about hypoglycaemia management in detail. Please elaborate on various causes of hypoglycaemia, the symptoms experienced when the blood glucose levels go below 70 mg/dl and how to manage hypoglycaemia. Refer to Chapter 4 in the "Diabetes in children and young adults" booklet.

3.1.10 Hypoglycaemia

Hypoglycaemia is defined as blood glucose levels under 70mg/dl. The various causes of hypoglycaemia have been enlisted in the slide.

- Missing a meal, or decreasing carbohydrate content of the meal, or delaying the meal after having taken insulin, can lead to hypoglycaemia.
- Another reason for hypoglycaemia is a mistakenly high dose of insulin. This can occur if the insulin strength is not matched to the proper syringe i.e. when 100 U/ml insulin vial is used with 40 IU insulin syringe and not a 100 IU insulin syringe. Similarly, if need be, always use cartridge insulin with a 100U/ml syringe.
- During exercise, muscles consume plenty of glucose. This effect can remain for 8 - 10 hours or more after the exercise is over (till the muscles replenish their energy stores) i.e. well into the night or early next morning. It is prevented by adjusting insulin dose and food intake. Try to remember to take the insulin injection in an area of the body which will not be used during exercising. Insulin enters the blood faster from an exercising limb and hence there may be greater reduction in blood glucose. To

prevent this, if you are using your legs while exercising like running or jogging, insulin can be taken in the arm or abdomen. If both arms and legs are used during exercise like swimming or playing hockey, then the injection before the exercise may be given in the abdomen.

- Vomiting and loose stools can lead to dehydration and loss of electrolytes. This can in turn can cause stress on the body and lead to increase in blood glucose levels.

Please elaborate on the various symptoms of hypoglycaemia as shown in the slide #9. The various symptoms of hypoglycaemia include trembling, weakness, irritability, hunger, sweating, anxiety, decrease in concentration during work, headache, inability to walk steadily and unconsciousness. Regarding the management of hypoglycaemia, please explain in detail about the several reasons using the information given as follows:

If it is possible to measure blood glucose, do so immediately. If blood glucose is below 70 mg or if there is no facility to measure blood glucose, then immediately eat glucose powder or sugar: 15 gm (3 tea spoon) for adults and 10 gm (2 tea spoon) for children. If you do not have glucose or sugar with you, 3-4 pieces of candy or 150 ml. unsweetened juice (or 100 ml of sweetened juice) will also help.

Stop all work and take rest for 10 minutes. After 10 minutes please test your blood glucose again. It should be above 100 mg/dl. Even if it is above 70 mg/dl the next step is have a snack consisting of 15 gm carbohydrate (eg. 1 roti or 1 cup milk, 1 large slice of bread, a large fruit or 3 biscuits), so that hypoglycaemia does not recur. If it is your meal time, take your usual insulin dose but eat your meal (instead of the snack) immediately instead of leaving the usual gap of few minutes. If blood glucose has not risen above 70mg/dl, take sugar or glucose again and repeat the cycle described above. Inspite of this if there is no improvement please consult a doctor immediately.

If there is severe hypoglycaemia you may not be able to take care of yourself. Somebody in the family can help you take glucose or sugar. Therefore, it is important to educate the family regarding hypoglycaemia management too.

In case of fits or unconsciousness, no water or food should be given. Glucagon injection should be given. It will take 5-10 minutes for the injection to take effect. After regaining consciousness, you must eat some food. Glucagon injection may produce vomiting; therefore, the child should be made to lie on his or her side so that no vomitus goes into the windpipe. Glucagon comes in a 1 mg. vial. Children below 12 years can be given 0.5 mg and those

above 12 years can be given 1 mg. It is given intramuscularly, into the muscle. If after giving sugar or glucagon injection no improvement is seen, or if you are confused whether you are giving proper treatment, then immediately call in the doctor, who can give intravenous glucose injection.

In case, the patient is unconscious and cannot eat or drink glucose or if glucagon injection is not available, you can make a paste of glucose, resembling toothpaste, by adding about 5 to 8 drops of water into 10-15 gm (2-3 tsf) glucose powder. Alternately, you can also use honey if glucose is not available. Take small quantities of the paste on your finger, pull open the lower lip of the patient, and apply on the oral mucosa in small frequent quantities, till all the paste has been applied over several minutes. At the same time, call a nearby doctor to come and give intravenous glucose. Once the patient regains consciousness, give a snack or meal. Buy a new glucagon without delay.

Once the episode of hypoglycaemia has been managed, look into / find the primary cause that had caused hypoglycaemia, to prevent future episode. Always keep a hypoglycaemia kit handy. We will discuss this in more detail in the later part of this presentation.

Slide #11

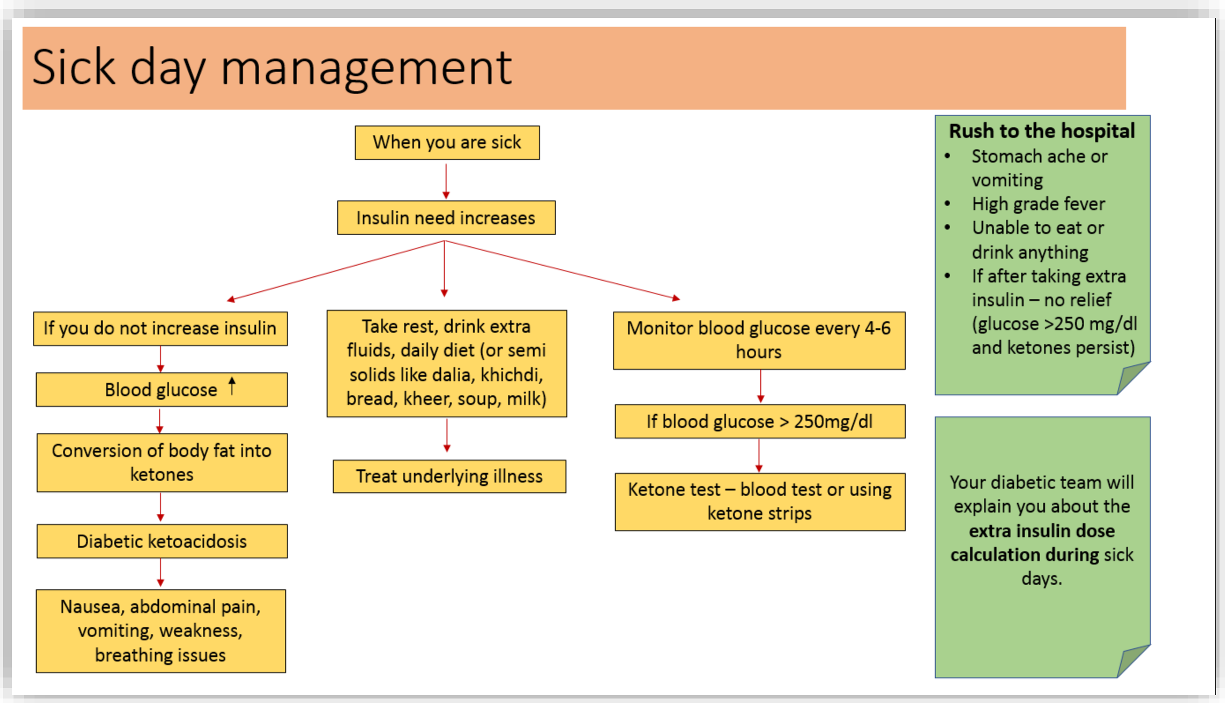

In the sick day management module, teach the participant about managing the fluctuation in blood glucose level when you fall sick. In addition, discuss about the reasons that lead to the patient falling sick. The biological causes, monitoring strategies and management of sick

days will be explained in detail. Refer to Chapter 7 and 8 in the “Diabetes in children and young adults” booklet.

3.1.11 Sick day management

Any type of disease, surgery or stress (including ordinary ailments like cold, diarrhoea, fever, toothache) can increase insulin requirement. When insulin is insufficient, blood glucose increases, there is excessive urination and there will be loss of fluid from the body. Subsequently, the body fat will be broken down to form ketones and in this condition of ketoacidosis, the patient can become unconscious. Diabetic ketoacidosis often called DKA is characterized by high blood sugar, vomiting, shortness of breath and positive ketones To prevent such a situation, people with diabetes should follow certain instructions at time of illness.

When you are ill you should take rest. You may take help from any member of your family for taking injections, measuring blood glucose and eating food. You should drink extra fluid (6-8 glasses) to prevent dehydration which can worsen ketosis. Other than water, you can also have tea, lemonade (without sugar) or lemon soda. If there is nausea, pain in the stomach or vomiting and you are not able to eat anything you should go to hospital immediately.

Keep up your daily diet. If you are unable to do that, then you can eat whatever is possible, in small frequent quantities. Dalia, khichri, bread with milk, kheer, ice-cream, custard, fruit, fruit juice, glucose biscuit etc. are some of the items which you can eat during illness every hour in small quantities. Continue your insulin injection daily during illness, even if you are not able to eat regular meals. Take some of the sweet items mentioned above. If you are not able to eat even these, then discuss with the diabetes team how to regulate insulin dose and proceed to the nearest hospital.

Blood glucose should be strictly measured every 4-6 hours. If blood glucose exceeds 250 mg, then please measure ketones in the blood or urine. For measuring this, use a strip called Ketodiastix or Optium blood ketone strip (if your blood glucose instrument is called 'Freestyle Optium'). If you do not have the strip you can get blood tested in a laboratory for ketones.

If glucose is high and there are ketones in the urine/blood, then you need extra dose of insulin. If the ketone level is mild or moderate and you can have 24-hour contact with the diabetes team, then you can manage at home. If ketones are high, or rise from mild to moderate despite extra insulin dosing at home, then you must get admitted in hospital. It should be always remembered that insulin should never be stopped during illness, rather

extra doses of insulin may be required during illness. Extra insulin should be taken only in the form of regular insulin (e.g. Actrapid, Insugen R, Huminsulin R) or rapid acting insulin (e.g. Humalog and Novorapid). During illness, calculation of each dose of extra insulin will be about 5-20 percent of your whole day's insulin dose. This extra amount of insulin is to be taken before meal or snack, in addition to the usual insulin taken at that time. Your extra dose of insulin will depend on the level of blood glucose and ketones.

3.1.12 Extra insulin dose calculation

An example to illustrate extra insulin dose calculation is as follows. If you take daily 6 unit regular + 12-unit NPH injection before breakfast, 8 unit regular in the afternoon, and 4 unit regular + 6 NPH before dinner, then during illness 5% extra insulin will be calculated as follows:

6 + 12 + 8 + 4 + 6 = 36 x 5/100 = 1.8 (or 2) units regular insulin.

This means if blood sugar is high before breakfast then you will take 6 + 2 unit regular and 12 units NPH. If it is before lunch, in the afternoon, then 8 + 2 unit regular should be taken and if before dinner, at night, 4 + 2 regular and 6 units NPH should be taken.

After 4 to 6 hours of taking extra insulin, you should measure your blood glucose and ketones. If blood glucose is more than 250 mg/dl or if ketones are still present, then more of extra insulin (e.g 10% or 20% of total daily dose) will have to be taken. If after all this, there is no improvement in your condition then consult your doctor. When blood glucose falls below 250 mg., then you may stop taking extra insulin.

Table 3.4: Extra insulin dose calculation

Blood ketones	Urine ketones	Quantity of Additional Insulin		
		Blood sugar less than 180	Blood sugar 180–250	Blood sugar more than 250
Less than 0.6 m mol/L	0 or Trace	Normal dose	Normal dose	5% of total daily dose extra
0.6 to 1.0 m mol/L	Minimum	Normal dose (this is starvation ketone*)	5% extra	5–10% extra
1.0 to 1.5 m mol/L	Medium	Normal dose (this is starvation ketone*)	5–10% extra	10% extra
More than 1.5 m mol/L	Moderate to high	5% extra	10 % extra	10-20% extra

3.1.13 Diabetic ketoacidosis

Ketoacidosis is a very serious condition. This occurs when the body does not get enough insulin. If diabetes is under control, then chances of developing ketoacidosis are low.

Ketoacidosis occurs when the required amount of insulin is not secreted in the body or is not available from the treatment. Reasons for relative lack of insulin are as follows:

1. Infection, injury, surgery or any other stress (during stress, the body requires more than the usual amount of insulin).
2. Not taking the full dose of insulin or if your insulin is expired or destroyed by heat. In patients using insulin pump, blockage/malfunction of pump can result in DKA.
3. Undertaking prolonged vigorous exercise on the background of inadequate insulin doses during the previous few days and/or prolonged high blood sugars.

The symptoms of diabetic ketoacidosis begin slowly and it often happens when the warning signs have been ignored for a few days. But in small children and young people symptoms may appear within a few hours. The signs of diabetic ketoacidosis include excessive urination and thirst, nausea, abdominal pain and vomiting, weakness, faintness (tendency to fall unconscious), fast breathing and drowsiness and unconsciousness (coma).

Slide #13

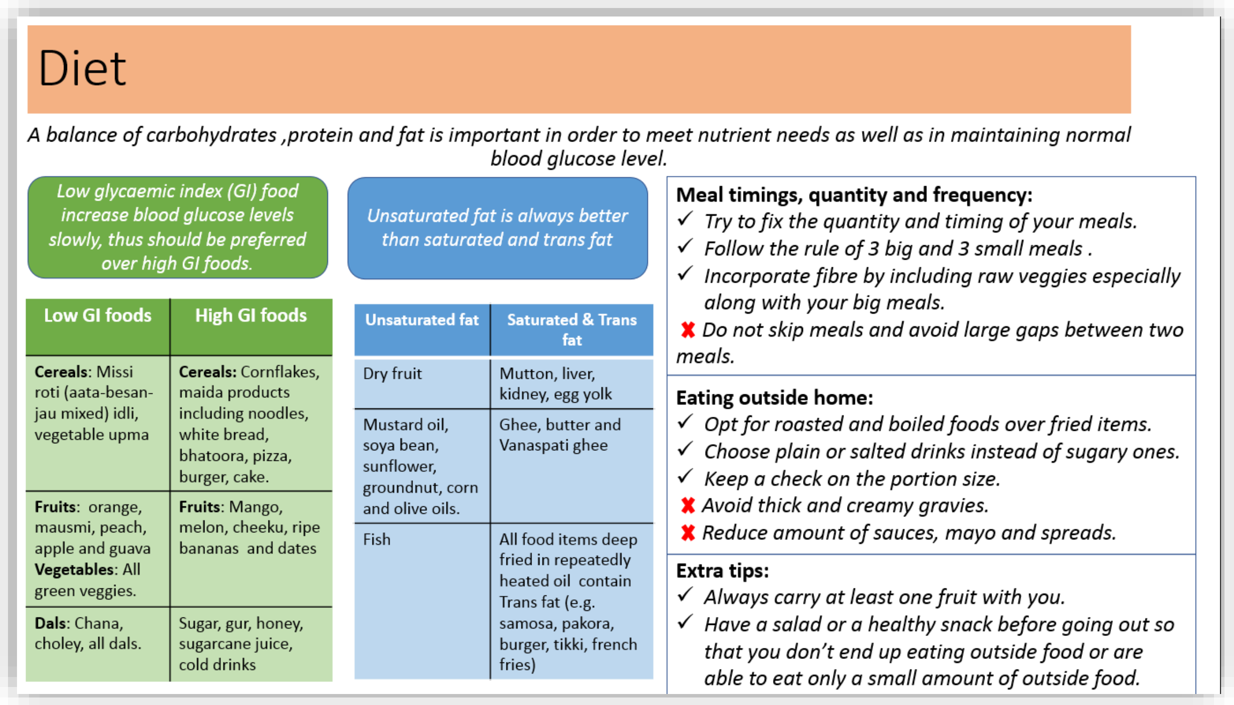

This slide contains detailed information on the diet to be followed in case of Type 1 diabetes (T1DM). The diabetes educator should explain to the participant the importance of a well-balanced diet, diet timings and meal portions. Refer to Chapter 3 in the "Diabetes in children and young adults" booklet.

Making wise decisions around eating is important for glucose control so that unstable blood glucose does not get in the way of work and play activities. A balanced diet is also important

for preventing cardiovascular disease, which people become more likely to develop as they grow older. Eating a heart healthy diet is even more important for people with type 1 diabetes who are 10 times more likely develop cardiovascular disease than people without type 1 diabetes.

Let’s review some of the eating tips that can help you protect your health now and in the future.

First let’s talk about choices that can help you manage your blood sugar. You might be familiar with low glyacemic index food and how they are helpful for maintaining stable blood sugar. This is because your blood sugar rises after you eat, but it rises more slowly when you eat low glycaemic index foods as compared to high glycaemic index foods, which can cause your blood sugar to spike.

When putting a meal together, choose missi, roti, idli, and vegetable upma, chana choley, and dal, over high glycemic index corn and maida products. What are some corn and maida products that you want to eat sparingly? Cornflakes, white bread, batoora, pizza, burger, cake.

Now how about fruits and vegetables? Fruits like mango, melon, cheeku, bananas and dates have high glycaemic index. So, while choosing a fruit to eat, try choosing low glycaemic index fruits like oranges, mausmi, peach, apple and guava. And as an easy rule of thumb, remember green veggies are always a great low glycemic index choice that also have many other health benefits!

One last tip for avoiding blood sugar spikes is to limit the consumption of sugar, gur, honey, sugarcane juice, and sweet cold drinks.

Now, we talked about how type 1 diabetes increases your risk of cardiovascular disease like stroke and heart attack, so let’s talk about replacing the saturated and trans-fat in your diet with unsaturated fat as much as possible, since unsaturated fat reduces your cardiovascular disease risk, while saturated and trans fats increase it. Whenever you can, swap out ghee, butter, and vanaspati ghee with mustard oil, soya bean, sunflower, groundnut, corn, and olive oil. Remember there is a high amount of saturated and trans fats in fried foods and sweets, so when you have a sweet tooth, try some fruit, and if you’re in the mood for something savoury, try a handful of roasted chana, puffed makhana, or nuts. Dark meats, intestines, and egg yolk have a large amount of saturated fat, so try to choose fish, leaner meat like chicken, and egg whites whenever possible.

Let’s review a few last important eating strategies that will help you with your glycemic control and cardiovascular health. Try to be as consistent as possible in the timing, quantity, and frequency of what you eat. Try to eat 3 meals and 3 snacks at roughly the same time each day and eat about the same amount, which will help you ensure that you match your insulin and food in a way that minimizes highs or lows. Some days you might be more hungry than usual, so try to eat green veggies and raw veggies like salad, which will help fill you up and not disturb your blood sugar as much as eating more of other foods. Plus the fibre in these green veggies is helpful for cancer prevention and maintaining a healthy heart!

We know it can be hard to eat in healthfully when you eat outside the home, so let’s review some quick tips for those scenarios when you are out with others:

To make sure your insulin can cover the food you eat, choose roasted and boiled foods over fried items. Choose plain or salted drinks rather than sugary ones, try to eat a portion size for that meal or snack that you usually eat, and avoid thick and creamy gravies. Use sauces, mayo and spreads sparingly. Remembering these tips will help make sure that what you eat outside the home matches your usual insulin dose as closely as possible and minimizes the risk of experiencing a blood sugar spike.

Because life can be unpredictable and when you go out with others you may not always be sure of the time you will be eating or what will be available to eat, it’s helpful to eat a salad or healthy snack before going out to eat, and always carry at least one fruit with you once you do. Adopting these practices will help minimize the risk of low blood sugar, as well as keep one from eating large amount of food that spikes the blood sugar because it is not diabetes friendly.

Any questions?

Slide #14

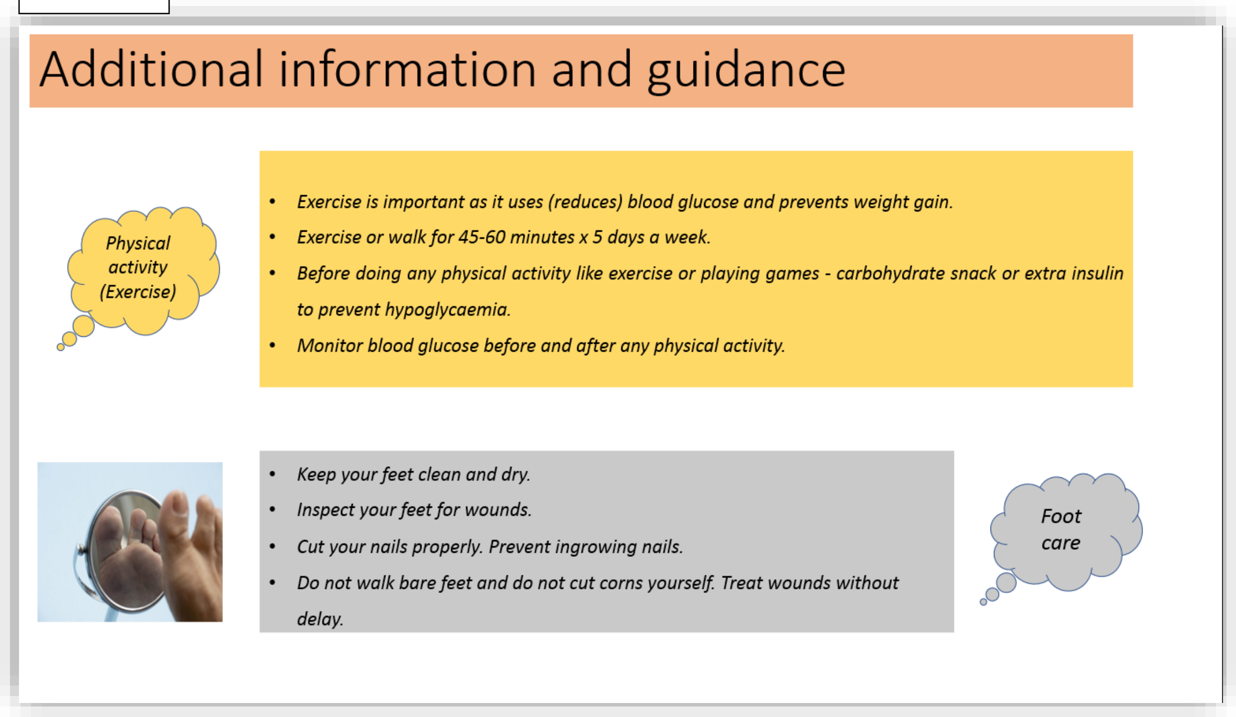

The slides #14, 15 and 16 aim to provide information on additional topics like importance of physical activity, foot care and pregnancy in diabetes. These short modules will also address issues like travel, career and living with diabetes. They should be discussed well and the participant should be made well aware of the importance of all these management matters and issues. Refer to Chapter 6, 9, 10, 11, and 12 in the "Diabetes in children and young adults" booklet.

3.1.14 Exercise

For good health it is essential to exercise every day. Exercise helps in strengthening the muscles, and keeping the heart in good condition. If you have diabetes then there are other benefits of exercise too. Regular exercise helps the body to utilize the glucose well. In this way, the glucose level in the blood decreases and it is easier to balance blood sugar. Exercise also helps in reducing the fat in the blood, which reduces the risk of high blood pressure, heart disease and stroke. Exercise also helps in reducing body weight in overweight/obese individuals, thus allowing the insulin to work better.

You should do only those exercises which suit you and your way of life. Daily exercise of 30-60 minutes will be good for you. If daily exercising is not possible then try for 5 days in a week. You should do exercises that involve increased use of energy. As for children their play is their exercise. Definitely if given space and friends, they would play. In addition, if you have the facility please encourage the child to excel in a certain game. Taking part in competitive sports is not prohibited. On the other hand, it will help in physical and mental development. Running, tennis, swimming, football, hockey, badminton, cycling, skipping, volley ball etc. and other such

games are all good. Yoga also makes the body active. Try to reduce the screen time to a maximum of 1 hour per day and dedicate extra time to physical activity or outdoor sports.

These were examples of aerobic exercises. The second category, i.e. anaerobic or resistance exercises include sit-ups and push-ups and weight lifting etc. A combination of aerobic and anaerobic exercise is ideal.

Table 3.5: Guidance before exercise/play based on blood sugar test result

Less than 90 mg/dl	Take 10 to 20 gm. carbohydrate, check blood sugar in 10 to 15 min., if it is more than 90 mg then you may go to play.
90 - 125 mg/dl	Take 10 gm. carbohydrate and then go to play
125 - 250 mg/dl	You may go to play without eating anything extra
250 mg/dl and above	Please check ketones and do not go to play if ketone is present. Contact your diabetes team. Take extra insulin as per instructions. If ketone is negative, you may play when the sugar normalizes.

Blood glucose may fall because of exercise. To prevent such a situation, you should eat a snack made of low glycemic index carbohydrate before exercise. You should measure blood glucose both before and after the exercise (Table 3.5). This will indicate whether the extra food eaten beforehand or the adjustment in insulin was sufficient to avoid hypoglycaemia. Try not to play at the time of maximum effect of insulin. While taking rapid acting insulin (Humalog, Novorapid), the effect is highest after an hour. If you are taking regular insulin (Actrapid, Insugen R, Huminsulin R) the effect is highest after 2-3 hours. If you have to play during such a time, then decrease that particular quick acting insulin dose by 25-50%. If you have not been able to take your dose, then take 10-20 gm quick acting carbohydrate (Table 3.6, 3.7). For every half hour of moderately vigorous play/exercise beyond the first half hour, you should take 10-20 grams of high glycaemic index carbohydrate snack. After finishing the exercise, take 15-30 grams of carbohydrate to replenish glycogen stores and avoid late hypoglycaemia. You may even need to decrease dinner time insulin by 10-20%.

Table 3.6: Examples of 15 gm carbohydrate exchanges which raise blood glucose quickly

Food Stuff	Quantity of carbohydrate
Bread 1 piece ~4 inches	15 gm.
¾ of big banana	13-15 gm.
Biscuit 3 piece (Parle G)	15 gm.
Juice 100 ml. (half of small pack)	15 gm.

Table 3.7: Grams of carbohydrate to be taken every half hour, during prolonged exercise

	Body weight 20 kg	Body weight 40 kg	Body weight 60 kg
Cycling at 10 km. per hr	8 gm.	12 gm.	18 gm.
Walking at 4 km per hr	8 gm.	12 gm.	15 gm.
Walking at 6 km per hr	12 gm.	15 gm.	18 gm.
Running at 8 km per hr	18 gm.	30 gm.	45 gm.
Basketball, football	15 gm.	30 gm.	45 gm.

3.1.15 Foot care

With diabetes, there is increased risk of wounds on the feet. This could occur due to various reasons: a) decreased sensation and function of muscles of feet caused by disease of nerves, b) increased dryness of feet caused by decreased function of sweat glands secondary to involvement of nerves, c) decreased blood supply preventing adequate delivery of oxygen and nutrition to the local area, and d) decreased wound healing and increased susceptibility to local infections. However, good care of the feet can prevent such wounds. You should wash your feet daily with soap and water and wipe them dry. If the skin is dry, apply cold creams or moisturizing cream. No spirit should be applied. If there is excessive sweating, use talcum powder but do not let the powder get caked between the toes. Inspect the feet daily for any wound. You can use a small mirror to examine the sole of your feet thoroughly for any cuts, fissures or callosities. It is also possible that due to decreased sensation, you may not feel pain on a wound and thus your attention will not be drawn to it.

Cut your nails carefully. Do not cut them too close. During winter, do not warm your feet close to a heater or fire. Use comfortable (not tight fitting) shoes and use cotton socks (preferably white colored to easily identify any blood soakage) without tight elastic Always buy a shoe in evening hours to account for any swelling caused by fluid retention that accumulates over the course of day. Never buy shoes with pointed tips, rather opt for well fitting, comfortable shoes with round toe box, sturdy outer sole and microcellular rubber (MCR) insole (where possible). Never walk barefoot even indoors. Avoid the use of hawai chappals, as they increase the risk of first web infection, rather opt for comfortable sandals with 2-3 straps and MCR insole. Treat wounds immediately with clean water and cover with

a clean cotton dressing. If it does not improve in 24 hours, meet a doctor. Never try to cut a corn on your foot yourself. Do not use 'corn cap' or 'corn remover'. Use Vaseline or moisturizer. If there occurs a corn on starting a new shoe which may be tight, change to a more comfortable shoe.

3.1.16 Treatment of wounds

1. Treat wounds without delay.
2. Wash the wound with clean, lukewarm water and Savlon®..
3. Apply ointment like Neosporin® or mupirocin. Don't use a strong irritant like betadine, mercurochrome, or carbolic acid, or "Band-aid."
4. Cover with a clean cotton dressing.
5. Do not use warm fomentation on a wound.
6. If it does not improve in 1-2 days or develops swelling, pus, redness, meet your doctor urgently.

Slide #15

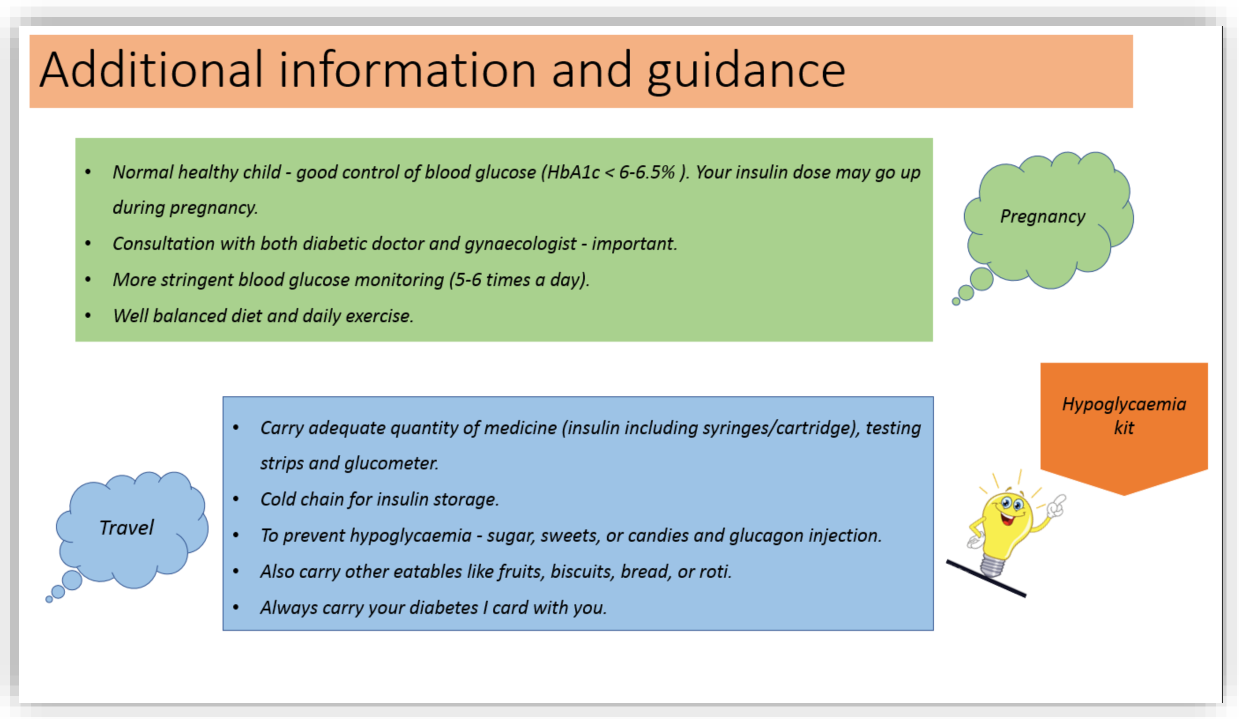

3.1.17 Pregnancy and diabetes

You can have a healthy baby even if you have diabetes before or during pregnancy. All you need to ensure is that take sufficient precautions and proceed with planning. Be in regular and close contact with your diabetes doctor and your obstetrician. It is important to remember eye (retina) and kidney problems caused by diabetes may progress or worsen during the course of pregnancy. Good control of blood sugar before and during pregnancy will help you to have a normal healthy child. It will also help in reducing problems during pregnancy and avoid developing diabetes complications in the eyes and kidneys.

Uncontrolled sugars during pregnancy, especially during the ‘first 3 months of pregnancy’ can cause harm to the organs of the growing fetus and may even cause some malformations. High blood glucose can sometimes result in abortion too. The baby's birth weight may be high; this can cause problems during delivery. The baby may have temporary hypoglycaemia after birth, and may develop jaundice. Later in life, the child or young adult may be at risk for diabetes. This risk is present even if the father of the child has diabetes, not just the mother.

Your blood sugar should be well under control for 2-3 months prior to pregnancy. The HbA1c should be between 6.0 to 6.5%. Do start taking folic acid even before you get pregnant. Consult an expert gynaecologist who has experience in looking after women with

diabetes. Do not forget to check the tests of long-term complications of diabetes, in addition to daily blood sugar checks.

3.1.18 Precautions during pregnancy

1. It is important to keep sugar under control. For this you might need to take insulin injections several times (3 to 5 times) a day. You should do a more stringent blood glucose monitoring i.e 5-6 times a day and keep a record. Lastly, adjust insulin dose according to your meal, appetite and blood sugar. Your insulin dose may go up during pregnancy.
2. You should eat a balanced diet containing proteins as well as calories in somewhat greater quantity than you did before pregnancy. You may take the advice of your doctor or dietician for this. Do not consume more of fatty foods. Women taking insulin should take a bedtime snack.
3. In addition, you should do daily exercise-walking, swimming etc., check for diabetes related long term complications 3 monthly. Get ultrasound or other check-ups done regularly and keep a watch on your weight and BP.

3.1.19 Diabetes and travel

Travelling is easy with diabetes; all you need to do is prepare well for the journey and do not forget to carry the essentials. The essentials primarily include medicines. Carry enough stock of insulin. If the weather is hot, then insulin should be kept in a thermos flask or in a thermocol box with an icepack. This is not necessary during winter months. If you are traveling by air, keep the insulin with you in the cabin, not in the checked-in baggage. Keep enough syringes and needles with you. Do not forget to carry the glucometer and sufficient glucose testing strips with you.

In order to avoid hypoglycaemia, always carry glucose (or sugar /sweet/candy/lozenges). In addition, you should keep other food (moderate glycemic index like fruit, biscuits and low glycemic index like roasted gram, sattu) for taking care of blood glucose for longer number of hours). If you are unable to get meals on time these may come in handy. Keep a bottle of drinking water also. If you are travelling alone, please let the fellow passenger or bus/train/aircraft staff know that you have diabetes, and what should be done for hypoglycaemia in case you are not arousable.

An ID card should be kept in the pocket saying that you have diabetes, what medicines you take and that if you are found unconscious or not behaving properly you should be taken to

hospital immediately. The card should contain your name, address, telephone number, doctor's name, dosage of insulin and names of other medicines.

Hypoglycaemia kit:

When you are away from home or travelling, always remember to take your Hypo Kit with you. It should contain glucose or powdered sugar, candy, some biscuits or other snack (fast acting sugars and slow acting sugars) glucometer with lancet and strips and a glucagon injection. In addition, it should also have your ID and diabetes identity card. Extra supply of insulin and syringes should also be kept.

Slide #16

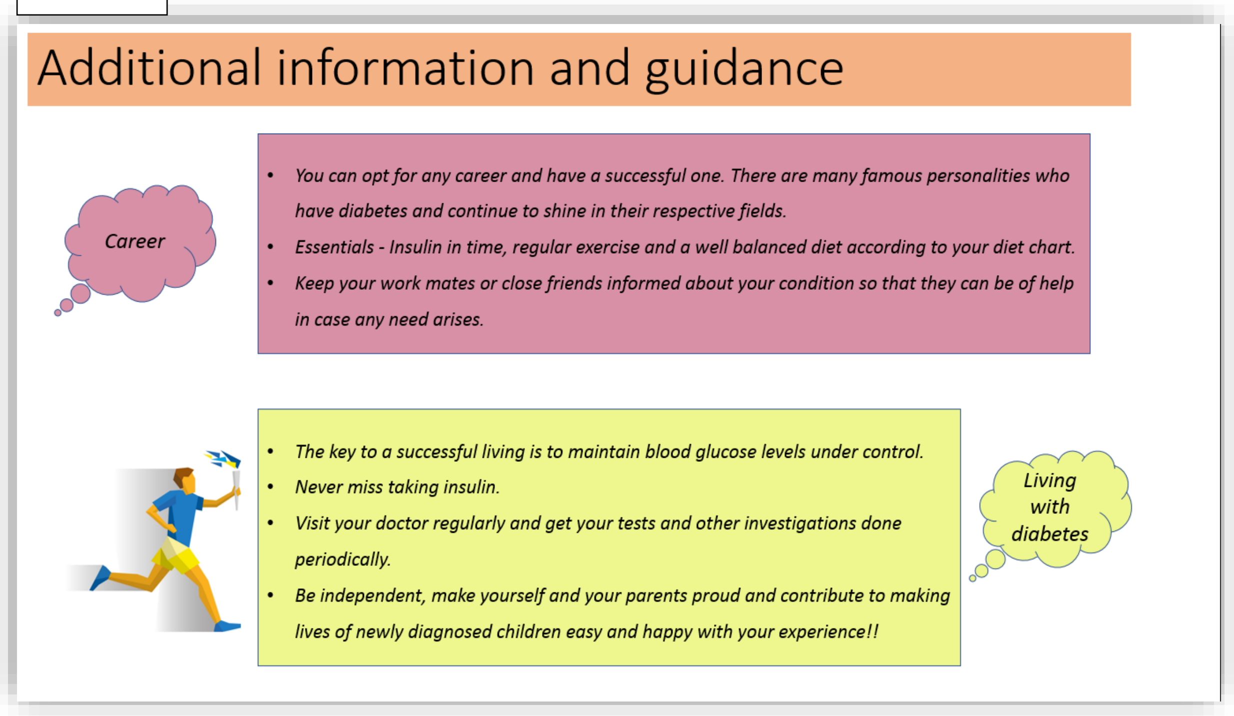

3.1.20 Living with diabetes

The diabetes team consists of the paediatric and adult health care provider, the dietician, the diabetes educator and most importantly you!! Diabetes as we now know is a health condition that affects large numbers across the world. Always remember it’s not you alone; its you along with many of your peers, people older than you as well younger to you. You can opt for any career as your heart desires for or you are passionate about.

Diabetes no way limits your capability to choose any career pathway.

If you now feel quite grown up, are in high school/ inter college/university and feel you are capable of looking after yourself, then your responsibility towards your health will also increase. You should continue to look after your health the way your parents used to when you were a child. You should tell your close friends about your diabetes, without any hesitation so that their help is available to you during times of need. If you are in a hostel and your parents have given you money for your daily needs, spend it wisely. If you have a doubt as to how to conduct yourself in any situation, do that which you feel would make your parents proud of you.

Basic Introductory Phase (Session 1) – Recommended adult provider list handout

S.No	List of adult endocrinologists	Details
1.	Name 1	i. Name of the hospital/clinic<br>a) Hospital ____________<br>b) Home clinic ____________<br><br>ii. Address of the hospital/clinic<br>a) Hospital ____________<br>b) Home clinic ____________<br><br>iii. Work timings/ days<br>a) Hospital ____________<br>b) Home clinic ____________<br><br>iv. Contact person<br>a) Hospital ____________<br>b) Home clinic ____________<br><br>v) Contact numbers<br>a) Hospital ____________<br>b) Home clinic ____________
2.	Name 2	i. Name of the hospital/clinic<br>c) Hospital ____________<br>d) Home clinic ____________<br><br>ii. Address of the hospital/clinic<br>b) Hospital ____________<br>b) Home clinic ____________<br><br>iii. Work timings/ days<br>b) Hospital ____________<br>b) Home clinic ____________<br><br>iv. Contact person<br>b) Hospital ____________<br>b) Home clinic ____________<br><br>v) Contact numbers<br>b) Hospital ____________<br>b) Home clinic ____________
3.		
4.		

3. Pre-Overlap Phase (Session 2) – Transition Rationale Reinforcement handout

Congratulations – you are soon ready to graduate to adult care!

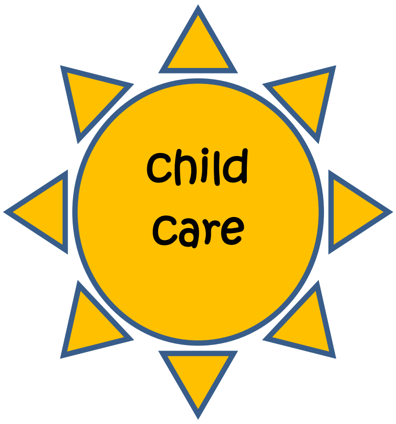

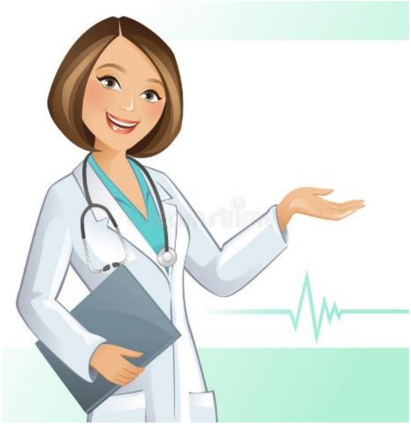

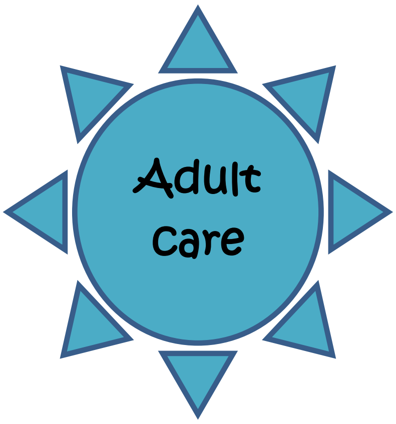

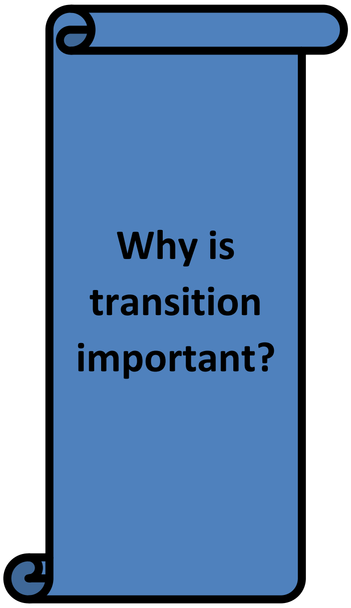

- *When you were a child you were being treated by a child doctor – paediatrician. As an adult, it is best for you to shift to a doctor who is specialized in treating an adult with diabetes i.e. Endocrinologist!*
- *Adolescence and young adulthood is a tricky period!! Your need for insulin changes, your hormones change, your body changes, where you work and live may change, and your behaviour changes; therefore, this unique time demands specialized care.*
- *The health system will not allow you to continue further with your paediatric doctor. You will face issues in getting the appointment, too.*
- *Lastly, transition is an important process through which we make you self-dependant in managing your diabetes as an adult.*
- *WE NURTURE YOU TO MATURE!*

Adult diabetes providers have the experience and training to help you most **happily** and **healthfully** navigate all that affects your diabetes as an adult:

College and Career

- *In college or when working, your commute time, mealtimes or locations (i.e., eating outside the home), and physical activity opportunities are likely to be more unpredictable than when you were in school. Adult providers can help you figure out strategies to take care of yourself amid these changes!*

Reproductive Needs

- *Your adult provider can help you with challenges or questions you have about your maturing body or about starting a family.*

Screening for and Managing Complications

- *Diabetes related complications become more common as you get older, so it is important to screen for them more frequently and have an adult provider who has the experience to help you manage them.*

Marriage and Family Responsibilities

- *Taking care of your diabetes while also juggling the needs and schedules of your family can be hard, but adult providers and patients have tips to help you!*

Insulin Needs

- *All of the physical and lifestyle changes you experience as an adult with diabetes means that your insulin needs will change as well. Your adult provider has the experience to provide ongoing*

Pre-Overlap Phase (Session 2) – Transition Roadmap handout

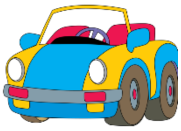

Namaste! Let me take you through the journey you are set to embark…

Session 1	Session 2	Session 3	Session 4	Graduation!

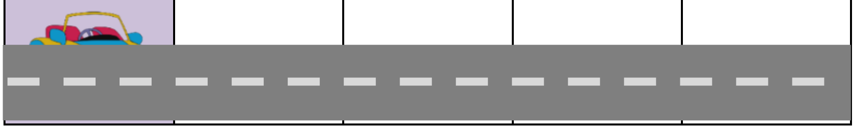

Session 1 (Paediatric Site): Today we shall discuss a few important things that you must know. You might know about all these already. However, I shall **refresh our memory** about diabetes and how do you take care of yourself and manage diabetes. We will also talk/discuss about a concept that was recently introduced to you; it is called **"Transition''**. We will discuss about it in detail like what is transition basically, why it is important and the need of transition.

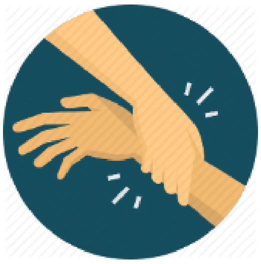

We will also discuss about what does **promotion in healthcare** mean. Did I tell you that you will be promoted in a few months' time from now?? In addition, I will give you tips on topics like diet, physical exercise, insulin management and any other topic that you would like to discuss.

After all, I am here to help you

Before I forget let me also tell you that I will introduce you to a **new universe of endocrinology and adult endocrinologists!!** Now who is an adult endocrinologist? Save your questions for me!!

*Also, you have to decide the name and/or place of an adult endocrinologist you would like to continue seeking treatment with. I promise I will **guide you in your decision** and in meeting your new healthcare provider.*

Session 1	Session 2	Session 3	Session 4	Graduation!

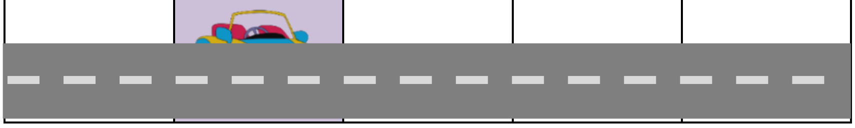

Session 2 (Adult Site): After 3 months when we meet, I will take you to the adult endocrinologist of your choice (Remember I had given you a list of adult endocrinologists the last time we met?? My work does not end here. I will also **introduce you** to the place and the people there. Cherry on the top, I will **make you meet** your new doctor – the adult endocrinologist! Any questions, please feel free to ask me.

Cherry on the top, I will **take you to meet** your new doctor – the adult endocrinologist! Any questions, please feel free to ask me.

Points to remember:

1. Do not shy away if anything is unclear or you are uncomfortable. Always ask questions and express concerns!
2. Be punctual. Never miss your appointments. Meet your doctor regularly.
3. Do not forget to get your routine tests/investigations done.

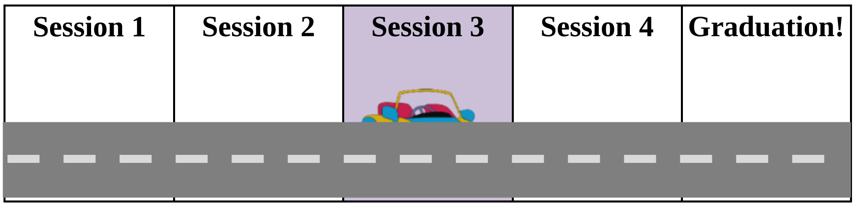

Session 3 (Paediatric Site): Now after next 3 months, we will meet at our old place again (our paediatric clinic)! We will discuss about your visit to the adult clinic. I want to know your first visit story and how did it go.

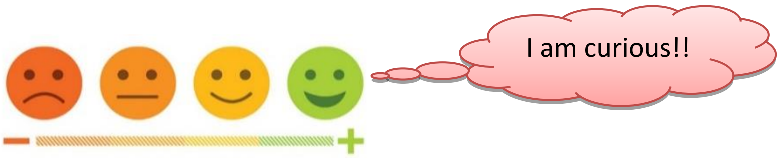

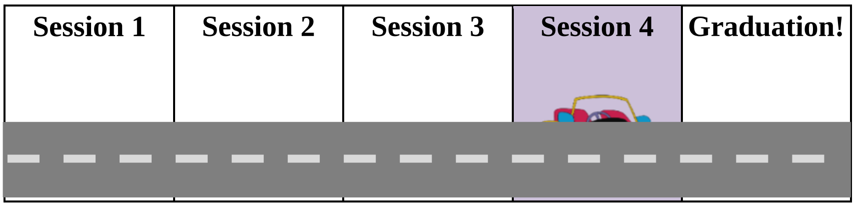

Session 4 – (Adult Site): After another 3 months, we again go to meet our new doctor – the adult endocrinologist. We make new friends there and realize that we are not alone. **There are others like me too.**

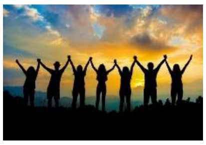

Would you like to hear their story?

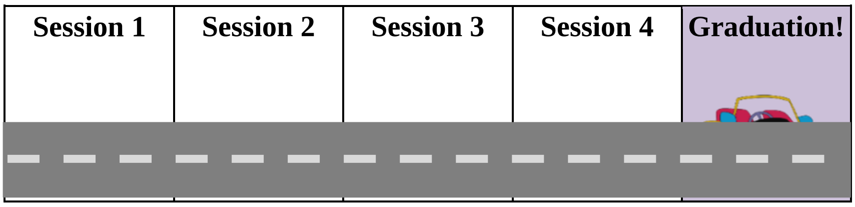

Graduation (Paediatric Site): ***Time for promotion!!*** You are ready to shoulder your diabetes care. So, it's time to get promoted to the next level in life. **Happy Graduation to you!!** You will be given a transfer package to help your adult provider take the best care of you. What does it contain? **That's a surprise!!**

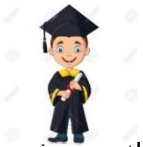

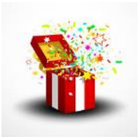

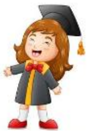

Stay in touch. We will meet again exactly one year after the day when you were transferred. That day, we will talk about how your journey has been. Then, I would like to meet you again after another year to share our experiences all the way through. Till then take care!

Pre-Overlap Phase (Session 2) – Adult provider commitment handout

1.	Name of the adult endocrinologist	
2.	Name of the hospital/clinic	Hospital:
		Home clinic:
3.	Address of the hospital/clinic	Hospital:
		Home Clinic:
4.	Work timings/ days	Hospital:
		Home Clinic:
5.	Contact person	Hospital:
		Home Clinic:
6.	Contact numbers	Hospital:
		Home Clinic:
7.	Any other information	

Patient details:

1.	Name of the patient	
2.	Age & Sex	
3.	Home address	
4.	Contact numbers	
5.	Name of father/guardian	
6.	Name of the physician presently seeking care with	
7.	Remarks	

Signature
(Diabetic educator/ treating physician)

Date:

Place:

Name
(Diabetic educator/ treating physician)

Overlap Phase (Session 3) – What should I expect from my adult provider team handout

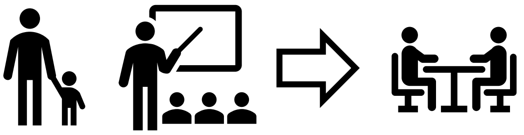

Now that you are an adult, making healthy decisions is **your** choice and responsibility. The adult provider team will work with you to help you make those healthy decisions, giving you a suggestion instead of an order. If you think a suggestion might not work, it is important to express that concern.

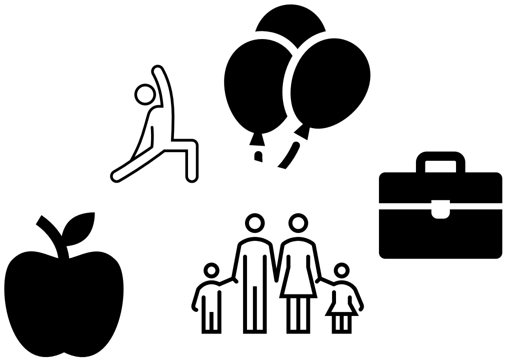

Every time you meet with your provider team, it is important that **you** bring up the situations when you find it hard manage your diabetes, so that the providers can help you improve your knowledge, skills and motivation to take good care of your diabetes while you're taking care of everything else in your life.

Remember that every adult with diabetes has a different job, family and social life, and schedule, so your adult provider team cannot give you the right care unless you tell them details about all of these things and keep them up to-date about any changes.

Be open and honest!

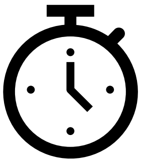

Not all adult provider teams have certain times set aside to just see patients with type 1 diabetes. Because they may see adults with other conditions like type 2 diabetes, you might find that you must wait longer to be seen. Remind yourself that every care you take now prevents costly complications later on…

…**waiting a bit longer now to live a healthier, longer life is worth it!**

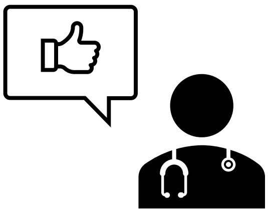

In times when your diabetes management is going smoothly for you, there may be shorter visits with the adult provider team then you are used to. This does not mean the provider is trying to shoo you away, so always feel free to bring up any concerns, needs, or struggles you may be facing so you **get the most out of your visit!**

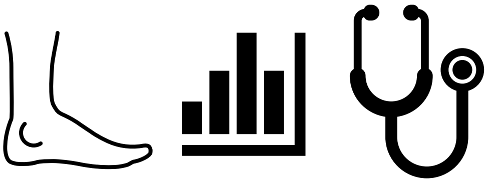

You should expect more screening for diabetes related complications in adult care. Remember that the more you participate in these screenings, the more your provider team can **help prevent or delay diabetes related health issues from getting in the way of your life.**

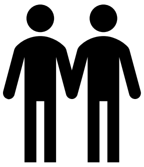

You might have more opportunity to interact with other adults with type 1 diabetes when you see your adult provider team. Adults with type 1 diabetes often have years of experience with the condition. They might have some healthy tips about managing diabetes as an adult and can help you realize you are not alone in the challenges that come with living with diabetes. **Take advantage of this opportunity to connect, but also remember what works for someone else may not work for you**, so check with your provider team before making changes based on what other patients do.

Overlap Phase (Session 3) – Eating to Thrive with Type 1 Diabetes handout

Eating to Thrive with Type 1 Diabetes

Why is it so important to think carefully about what I eat when I have type 1 diabetes?

You have probably been told before that it is important to **match *what* and *when* you eat to the dose and time of your insulin injections.** Doing this prevents hypoglycemia and hyperglycemia that can lead to frightening consequences like DKA or nighttime hypoglycemia.

In addition to making choices about food timing and quantity to keep your blood sugar stable, it is important to choose **higher quality** foods (*we'll get to this shortly!*). This is because type 1 diabetes makes you more likely to develop health complications and you are more likely to develop them earlier in life than people without type 1 diabetes. Eating in a diabetes-friendly way ***now*** prevents health problems that will burden you from living out the dreams you have for your life!

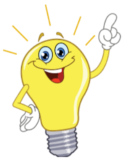

Sometimes it's hard to eat in a diabetes-friendly way. But remember that on days when you don't, hyperglycaemia and hypoglycemia get in the way of the activities you want to accomplish.

Over time, these days add up to diabetes complications, which will get in the way of the things you want to accomplish when you're older!

Keep these facts in mind when you are making decisions about when and what to eat!

What is a diabetes-friendly way of eating?

You may have been counseled that certain choices around food quality, quantity, timing, and spacing are more friendly to diabetes than others. ***Let's review together!***

So what foods are higher "quality"?

You may remember that the total amount of food you eat in a day should be made up of a balance of nutrients. These are carbohydrates, fat, and protein.

When you think about all of the food you consume in one day, about half should be from carbohydrates, about one third from fat, and the remainder from protein. However, certain foods have carbohydrates, fats, and proteins that make them healthier for your diabetes than others. This means that to ***thrive*** with diabetes, you want to include these foods as much as possible in what you eat throughout the day.

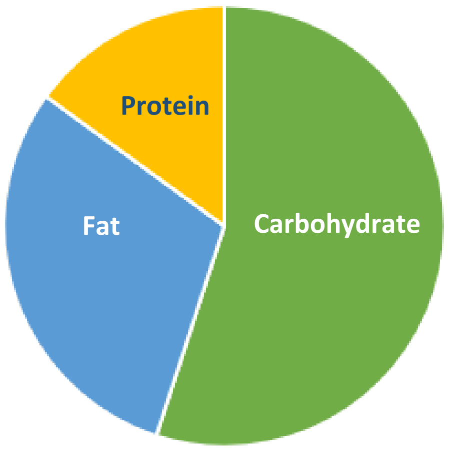

A full day of energy from food

Let's refresh our memories about these higher quality foods and how to combine them into a healthy plate!

Carbohydrate

Carbohydrates make up a little more than half of the energy in your diet! They also have the biggest impact on your blood sugar levels, but certain carbohydrates like those in sweets and root vegetables make your blood sugar rise faster and more than carbohydrates like those in whole grain foods.

Incorporating foods that do not make your blood sugar rise as much after meals –**high fiber** and **low glycaemic index** (GI) foods-- may help you meet blood sugar targets.

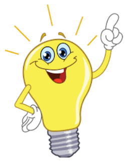

Fibre is the part of food that makes you feel full, prevents constipation, slows digestion, and helps keep your blood sugar from rising quickly

The glycaemic index ranks foods based on how they make your blood sugar rise 2 hours after you have eaten them

Which foods are high in fiber?

Whole fruits
- Apples
- Pear
- Papaya
- Prunes
- Citrus
- Watermelon

Seeds

Nuts

Beans

Lentils

Vegetables
- Green beans
- Dark green leafy vegetables
- Isabgol
- Peas
- Cucumber
- Tomatoes

Oat bran

Barley

Whole grain bread and cereal

Which foods have a low glycemic index?

When choosing between foods, select more **green** foods (low GI) in your diet than **yellow** (moderate GI). Eat **red** (high GI) foods most sparingly.

Brown rice	Basmati rice	White bread
Oats	Honey	White rice
Barley	Popcorn	Puffed rice
Bajra	Ice cream	Jowar
Buckwheat	Dried rice noodles	Ragi
Whole wheat bread	Black gram	Maize
Beans	Green gram	Semolina
Pulses	Croissant	Tapioca
Lentils		Cornflakes
Soybeans		Arbi
Peas		Potato
Spinach		Juice
Kale		Soda
Mushroom		Jam or Jelly
Cauliflower		Dried fruit
Kidney beans		
Cashew nuts		
Strawberries		
Apple		
Orange		
Low fat milk		
Yogurt/Daliya		

Quick carbohydrate tips!

- Select high fiber and low glycemic index foods whenever possible
- Eat the skins of fruit and vegetables (high in fiber!)
- Eat a least 2-3 servings of high fiber, low glycemic index fruit and vegetables per day
- Eat whole fruit instead of drinking juice because of the high glycemic index and low fiber of juice
- Eat wholegrain varieties of bread instead of those made with white flour, but remember porridge and whole wheat chapati have more fiber than even whole grain bread, so select these instead when possible!
- Do not consume bread dipped in tea or milk, eat with vegetables instead (i.e., cucumber, tomato sandwich)
- You can dampen the post meal spike in blood sugar caused by a high glycemic index food like rice by:
  - Adding fiber (i.e., add a lot of vegetables to rice or poha while cooking, and consume a greater portion of dal/sambhar/rasam than rice)
  - Eating a full plate of salad before eating anything else
  - Using brown rice instead of white rice

Fat

You may have heard that some fats are healthier than others. Maybe you have heard that you should eat saturated fat sparingly, which is the fat in butter, ghee, palm oil, whole milk and cream, desserts, and dark/red meats. Having type 1 diabetes makes you more likely to develop cardiovascular disease, heart, and stroke, which makes reducing the unhealthy fats in your diet so important. More diabetes friendly fats (polyunsaturated, monounsaturated) can be found in:

Seeds (i.e., flaxseed, sesame)

Poultry without skin

Nuts (walnuts, almonds)

Olives

Rice-bran

Peanuts

Avocados

Eggs

Fish

What about cooking condiments?

Choose

Safflower, sunflower, soya, cottonseed, corn, peanut, groundnut, mustard, and sesame oils

over…

Butter, ghee, cream, palm oil, coconut oil

Limit to 1 spoon per day!

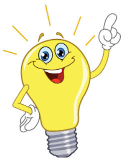

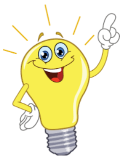

Vary the oil you use for cooking to get a balance of healthy fats

What if I have a sweet tooth, or am at a party or celebration where there are sweets served?

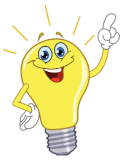

Ask your dietician about how to fit the occasional sweets into your diet, and how to safely plan for social celebrations or meals taken outside the home!

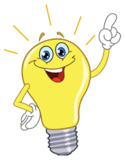

Let's review some quick fat tips!

- Use toned milk instead of full cream milk
- Use fish or lean meat instead of red meat
- Remove visible fat from meat (skin from chicken)
- Eat grilled, boiled, and steamed food instead of fried food
- Avoid dalda, vanaspati, fast food, and street food (tikki, samosa, pakoras)
- Change cooking oil at least every fortnight

Protein

Remember we mentioned saturated fat is not friendly to diabetes? Well, some protein rich foods are also high in saturated fat (i.e., dark meats, poultry with skin, whole milk and cream), so incorporate more of the friendlier proteins into your meal plan:

Fish | Eggs | Low-fat milk or yogurt | Soy | Beans | Lentils

How should I split up the food I eat throughout the day?

A dietician may have given you a meal plan that includes 3 main meals or may have given you a meal plan that includes 3 main meals, two mid-meal snacks (1 between breakfast and lunch, 1 between lunch and dinner), and bedtime snack (i.e., a half cup or full cup of double tone milk). This is designed so that your blood sugar levels coordinate properly with the levels of insulin in your body throughout the day to avoid hypoglycemia or hyperglycemia.

How should I time my food?

This varies person-to-person based on what insulin regimen they are on. If you are on a fixed-dose insulin regimen, you have been told to eat each meal and snack at roughly the same time each day, and administer insulin 20-30 minutes before certain meals or snacks.

You may have also been told to leave a 6-hour gap between main meals, and wait at least 2-3 hours after a meal before eating a snack.

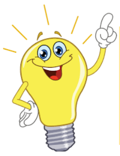

If you have not visited with the dietician in 6 months, it's time to take a visit to make sure you are eating in a way that helps you reach your height potential and prevent diabetes complications!

What is an example of a day's schedule of diabetes-friendly food on a fixed-dose insulin regimen?

The below table and pictures illustrate healthy food timing, spacing, and types of healthy foods. Because people are different sizes and have different physical activity routines, the amount of food a person should eat each meal varies. ***Talk to your dietician about the amount of food that is right for you!***

**Meal**	**Time**	**Food**	**Other Options**
Breakfast	8-8:30 am	1 glass of milk and 2 roti	2 brown bread; 1/3 cup of daliya and vegetables
Snack	10:30-11 am	1 pear	1/3 cup of sprouts or roasted channa; 1 medium size high fiber, low glycemic index fruit
Lunch	1-1:30 pm	Salad (carrot, radish, cucumber, tomato, onion), dal, vegetable, curd, 1 ladle of rice, 1 roti	Have two roti instead of rice + roti
Snack	5-5:30 pm	Tea and 1/3 cup roasted channa	Tea and 20 almonds; tea and 1/3 cup sprouts
Dinner	8-8:30 pm	Salad (carrot, radish, cucumber, tomato, onion), dal, vegetable, curd, 1 ladle of rice, 1 roti	Have two roti instead of rice + roti
Snack	10-10:30 pm	1 glass of milk	1 medium size high fiber/low glycemic index fruit; 1 cup of curd

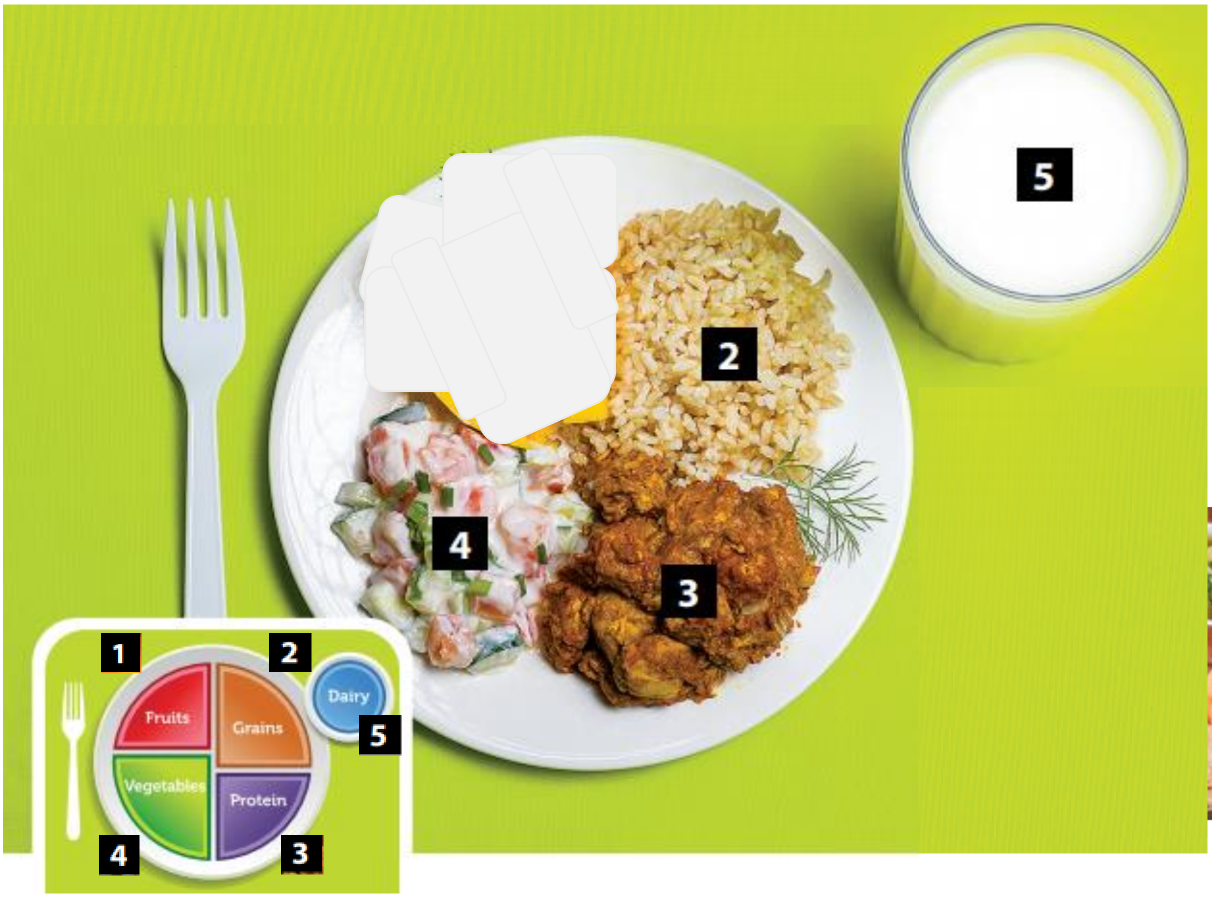

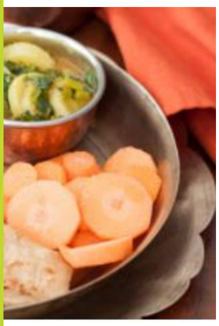

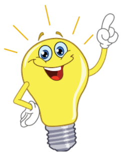

Remember that unlike sugar, salt, or oil, using herbs, lemon juice, vinegar, spices, onions, tamarind, and green pepper can enhance the flavor of your plate in a *healthy* way!

In sum, to keep your blood sugar levels stable, **it is crucial to be consistent about the way you have been counseled regarding the time you eat and the amount you eat, as well as the time and amount of insulin you inject.**

This is because you need your blood sugar levels to coordinate properly with the levels of insulin in your body. When you disrupt this coordination through food or insulin injection decisions, your life will be disrupted by hypoglycemia or hyperglycemia.

Of course, we understand that coordinating food and insulin in the way you have been counselled can be very challenging, especially with an unpredictable daily schedule.

Fortunately, there are skills you can learn to overcome these challenges!

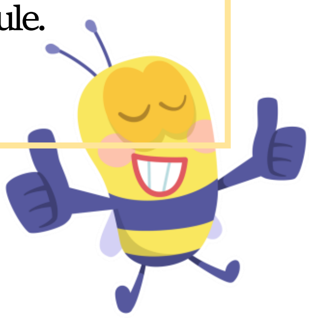

These skills are called **problem solving** and **"SMART" goal setting**.

We will build these skills over the next few sessions so that you feel comfortable using them on your own to overcome the obstacles to diabetes self-care you face now and the obstacles you will face in the future when your work, family and social obligations may change in an unpredictable way.

Overlap Phase (Session 3) – Problem Solving and Goal Setting handout

Problem Solving and Goal Setting

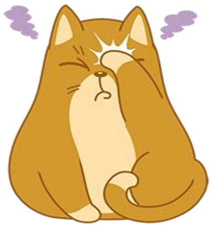

Sometimes you find yourself unable to do all the things you have been advised to do to take care of your diabetes –eating when and what you should, taking insulin in the proper way, checking and logging your blood sugar, exercising, visiting the clinic when you should, and getting routine tests done.

Sometimes things do not always go as planned because of school, work, family responsibilities, or social activities, which might throw off the diabetes routine you have been taught!

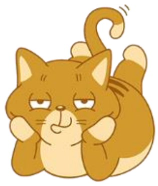

Sometimes diabetes feels like a full-time job and you want to forget about it for a while, so you don't care for yourself as you know you should! In the moments when your blood sugar is too high or low, you may become frustrated or not motivated to take care of your diabetes at all for a while.

This is where **problem solving** and **"SMART" goal-setting** skills can help!

Did you know that people with type 1 diabetes who learn these skills report **better health** and are **happier** than people who do not?

I want to know more! But first, what is problem solving and SMART goal setting, exactly?

This will become clearer in the next activity we do together, I promise! But briefly,

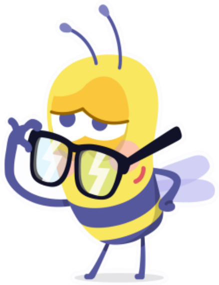

<u>Problem solving</u> helps you look at all the **reasons** that might have led you to have unstable blood sugar, or might have made you frustrated about your diabetes, or might have made you to be not motivated to take care of yourself.

Making <u>"SMART" goals</u> helps you think up **strategies** that are likely to prevent this bad situation from happening next time. Once you decide on the strategy, you then test it out in real life. You see whether it works or not. If the strategy does not work, you problem solve by asking yourself "Why?", and then you make another, better "SMART" goal to try again!

Over time, you discover the strategies that work, and you become more and more of a master in taking care of your tricky diabetes!

Why does problem solving and goal setting work?

Well, everyone has different bodies, different personalities, different social support, and different environments in which they care for their diabetes. This means everyone's ***obstacles*** to taking care of their type 1 diabetes are different and everyone's ***solutions*** to overcoming those obstacles will be different.

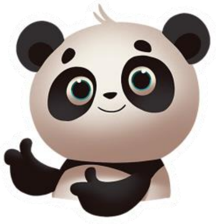

Through problem solving and SMART goal setting, you learn how to stop repeating the same actions that end up making you feel bad, and learn the actions that make you feel good!

Why is it important to start practicing now?

Learning these skills is especially important as you transition into being an adult with diabetes, because you will be more successful in taking care of diabetes without relying as much on your family or doctors.

Knowing how to problem solve and set SMART goals, you will be better able to adapt to the unpredictable situations you will face in adult life that will challenge your diabetes control and health.

This is because you will be able to identify successful self-care strategies more quickly and independently.

So, let's give it a try!

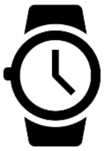

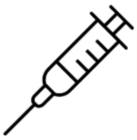

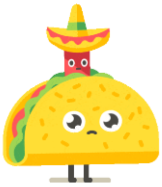

Let's think back to the topic we just discussed:
following a diabetes friendly diet.

We talked about how this involved making choices about food amount, quality, and timing of eating, and coordinating these choices with your insulin injections.

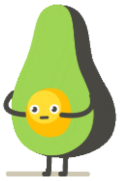

Take a minute to think about biggest problems you face in making these choices according to the way you are counselled.

Overlap Phase (Session 3) – Problem Solving and Goal Setting Activity #1 handout

Problem Solving and Goal Setting Activity #1

What is an obstacle you face in following a diabetes friendly diet right now? What causes the most concern or is most worrisome for you or your family?

Answer: ____

What worked?

What did not work?

START HERE!

When does it happen: __________________

Where does it happen: __________________

Who else is involved: __________________

Why does it happen: __________________

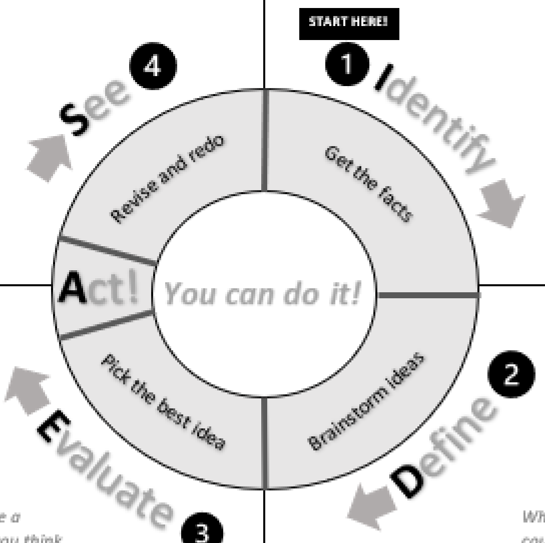

Flip over the page and make a SMART goal with the idea you think will help the most

What is one (or more!) thing you could do that might help improve the problem?

Specific
Exactly what will I do? When? Where?
Who will be involved?

Measurable
How much will I do (i.e. amount, minutes)?
How often?

Achievable
On a scale of 0 to 10, how confident am I that I'll be successful?
If less than 7, how can I adjust my plan so that I'll be successful?

Relevant
Why is doing this important to me?

Time-bound
When will I make my first step?
How long will I try this for before reviewing and updating my plan?

The biggest things that might keep me from success:

What I can try to overcome them:

SMART goal

Starting on ____________ *(day), I will* ____

____ *(what/how much)*

____ *(where/when/how often).*

I will try this until ________________________ *(day) before reviewing and updating my goal.*

Overlap Phase (Session 4) – Adult challenges for diabetes self-management handout

There are certain scenarios many adolescents and adults experience that can make it hard for them to prioritize diabetes self-management and be in good control.

Which of the following situations do you **currently** find challenging for managing your diabetes or do you think you will find especially challenging **in the future**?

Higher Education

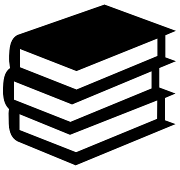

- Pressures of academic achievement compete with diabetes self-care
- Varying commute times throw off diabetes self-care routine
- 2–3-hour gaps between lectures means unpredictable social opportunities for physical activity or eating outside the home
- Managing food choices outside the home and varying meal times
- Unpredictable times or physical spaces to conduct self-management tasks (i.e., food, insulin, blood sugar monitoring)
- Addressing discrimination from teachers and peers or misunderstandings about diabetes
- Managing a diabetes self-care routine when living independently or with roommates in hostels
- Not wanting to disclose diabetes because of stigma
- Dynamic daily schedule due to day-to-day differences in class, study obligations, and social opportunities

Work Force

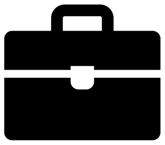

- Discerning which jobs might prevent you from taking care of your diabetes properly, and which jobs your diabetes might challenge you from doing the work properly
- Pressures of career promotion compete with diabetes self-care
- Dynamic daily schedule due to fluctuating work requirements
- Challenges finding time or location for exercise
- Varying commute times and work length throw off diabetes self-care routine
- Unpredictable times or physical spaces to conduct self-management tasks (i.e., food, insulin, blood sugar monitoring)
- Addressing discrimination from superiors and colleagues or misunderstandings about diabetes
- Managing a diabetes self-care routine when living independently or with roommates in hostels

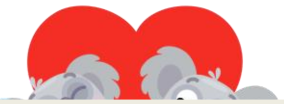

Marriage and Family Life

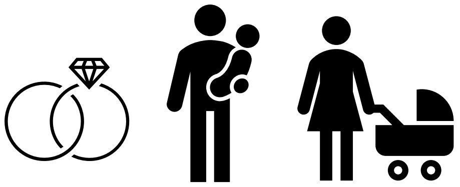

- Difficulty finding a partner
- Not sure how or whether to disclose diabetes to potential partners or their families
- Managing a diabetes self-care routing in a different home environment and/or different diabetes social support
- Family member discrimination or misunderstanding about diabetes
- Sexual dysfunction
- Children may be harder to conceive
- Health of offspring
    - *Your children may be more likely to have metabolic issues like overweight, hypertension, and diabetes, making it extra important that the entire family follow a healthy lifestyle to help prevent these health issues*
- Family advice that conflicts with doctor
- Arguments or scolding about diabetes
- Challenge to balance others' needs with self-care
- Navigating spouse, parent, and/or in-law roles in diabetes care

Friends/Peers

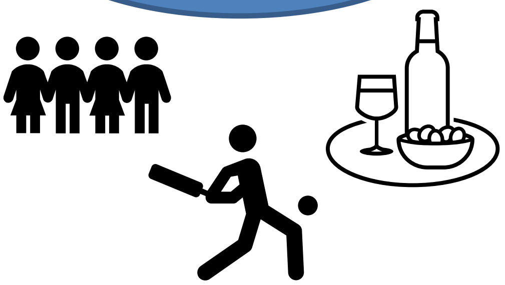

- Wanting to fit in to an activity (i.e., outing, sport, meal) competes with diabetes self-care routine
- Addressing friend discrimination or misunderstanding about diabetes
- Not wanting to disclose diabetes for fear of stigma
- Friend advice that conflicts with doctor
- Smoking
    - *Remember that having type 1 diabetes already puts you at high risk of cardiovascular complications (heart disease, stroke). Smoking speeds up complication development.*
- Drugs, Alcohol
    - *Remember these make you more likely to experience severe hypoglycemia, DKA, and higher HbA1c, which increase likelihood of complications now and when you are older.*

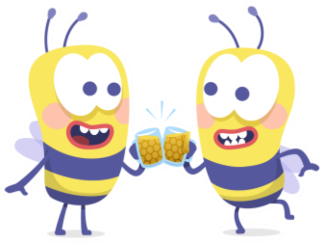

Mind and Mood

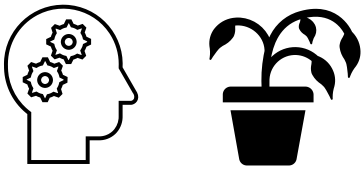

- Feeling anxious or depressed
- Feeling hopeless about the future
- Not being able to fall asleep or stay asleep
- Not eating what and when would keep my diabetes better controlled
- Not wanting to follow health provider advice
- Not seeing any reason to manage my diabetes

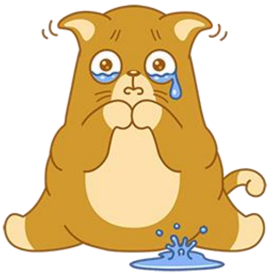

Remember that the more you work on managing your diabetes **now**, the more you will be thankful **later** when complications from diabetes do not keep you from achieving the goals you have for your life!

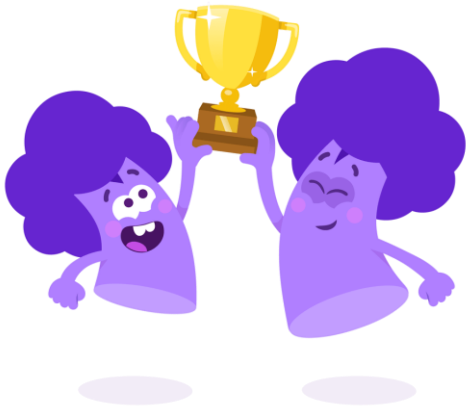

So, let’s talk about a challenge you are facing and choose one to work on!

Overlap Phase (Session 4) – Quick tips for common diabetes self-care challenges handout

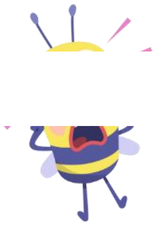

College, job, family and social obligations can make for a *dynamic* schedule. This unpredictability creates challenges for coordinating food and insulin, monitoring your blood sugar, and exercising in the way you know will keep you healthy **now** and into the **future**!

Here are some quick tips to get ahead some of the challenges that come with a dynamic schedule!

- Keep a hypoglycemia kit and blood glucose monitor in your bag at all times. Hypoglycemia kit should include:
  - Short acting carbohydrate source (i.e., glucose powder/tablets, powdered sugar, sugar candies)
  - Long-acting carbohydrate source (biscuits, bread)
  - Glucagon injection
  - Blood glucose meter
  - Glucose strips
  - Lancet
  - Lancing device

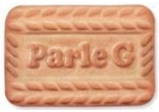

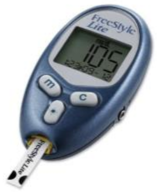

- In addition to your hypoglycemia kit, carry some low glycemic index, high-fiber carbohydrate foods in case you have to delay a planned meal or snack because of an obligation, or if you feel pressure to join peers for a snack at the canteen or other outside food establishments. These will prevent you from going into hypoglycemia, while also avoiding blood glucose spikes. Examples include:

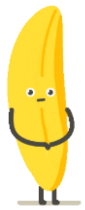

  - roasted chanas
  - nuts
  - makhanas
  - apple, orange, guava, medium size banana
  - vegetable sandwich with multigrain bread

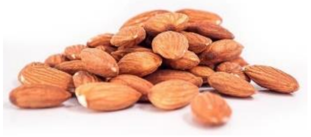

- If you partake in physical activity, monitor blood glucose prior to starting, check it during, immediately after, and 1-2 hours after you finish

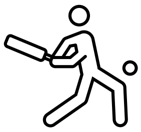

  - Do not start physical activity if blood glucose <100 mg/dl or >250 mg/dl with positive ketones. Avoid exercising during peak action of insulin (i.e., 2 hours for regular insulin)
  - Eat a snack before starting physical activity of at least 30 minutes, eat 15 grams of carbohydrate, such as 1 piece of bread, 1 moderate size banana, 3 biscuits (i.e., Parle-G)
  - For every hour of moderate/vigorous exercise beyond the first 30 minutes, eat an additional 10-20 grams of carbohydrate
  - After exercise, eat 15-30 grams of carbohydrate to avoid late hypoglycemia (i.e., 1-2 slices of bread, 1-2 medium bananas, 3-6 biscuits)
  - To help avoid nighttime hypoglycemia, do not exercise within 2 hours of sleeping

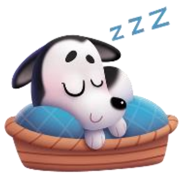

- Carry home-cooked food as much as possible, since you are better able to predict the effect it will have on your blood sugar than you are able to with outside food. When you must purchase food outside the home.

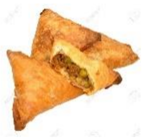

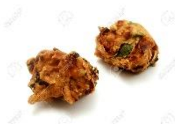

- Avoid eating junk food, fried food, or ready-to-eat products such as chowmein, noodles, pakoras, samosas, kachori, burgers, pizza, and french-fries

- Select low glycemic index and high fiber foods such as chapatis and vegetables, mostly vegetables with a little rice, and dal/sambhar/rasam

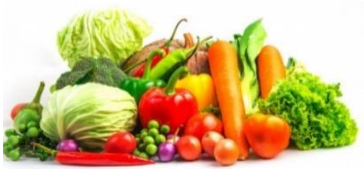

Consider ordering a proper meal containing roti/rice, dal and vegetables, idli/sambhar, vegetable upma/sambhar, plain dosa/samhar, multigrain bread vegetable sandwich

- Your glycemic response will be different, even if the dish is the same as what you eat at home, so it is essential to monitor your sugar more carefully. You may have to alter the pre-meal insulin dose for days you consume food outside the home.

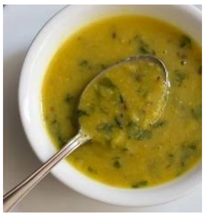

Unpredictable long working hours followed by long travel time might mean you will miss dinner at home some nights. Carry dinner from home as much as possible, but also identify a back-up plan in case this is not always feasible. Identify a shop with a meal similar to what you normally eat for dinner and plan to eat there on busy days. Maintain the portion and timing as similar as possible to what you would have eaten when at home.

- Depending on how far college or work is from your home, you may have to leave home very early. You may have to shift your breakfast and morning snack to a different time than you did at school, but try to eat them at the same time each day.

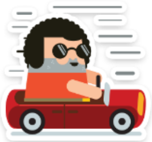

- Once you have a better sense of what your schedule will be in college or at work, meet with the dietician so that you can accomplish the activities you set out to do each day without being disrupted by avoidable hypoglycemia and hyperglycemia.

- If you fear stigma from conducting self-care in public, identify a consistent place for administering bolus insulin shots and testing your blood sugar. ***Never*** inject insulin through your clothing as this causes infections.

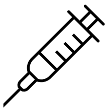

- You might fear that disclosing your type 1 diabetes will affect your social, academic, or career prospects. If you do not feel comfortable disclosing upfront with a new peer group or at a new job, overtime you may be able to identify an ally with whom you have built rapport and feel comfortable disclosing. Start with people you trust and grow from there! To prevent misunderstandings, consider emphasizing:

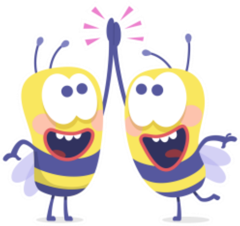

  - Type 1 diabetes is not contagious, it just means my body does not produce the insulin our bodies require to absorb the food we need to live, so I need to take insulin injections and maintain a healthy diet and physical activity schedule
  - If I keep on top of these diabetes care activities every day, diabetes will not get in the way of my academic or professional progress
  - Type 1 diabetes is not the same as type 2 diabetes and requires different care to stay healthy

Overlap Phase (Session 4) – Problem Solving and Goal Setting Activity #2

Problem Solving and Goal Setting Activity #2

Think about the scenarios we just reviewed: what situation do you find especially challenging for managing your diabetes?

Answer: ____

What worked?

What did not work?

When does it happen: ____________________

Where does it happen: ____________________

Who else is involved: ____________________

Why does it happen: ____________________

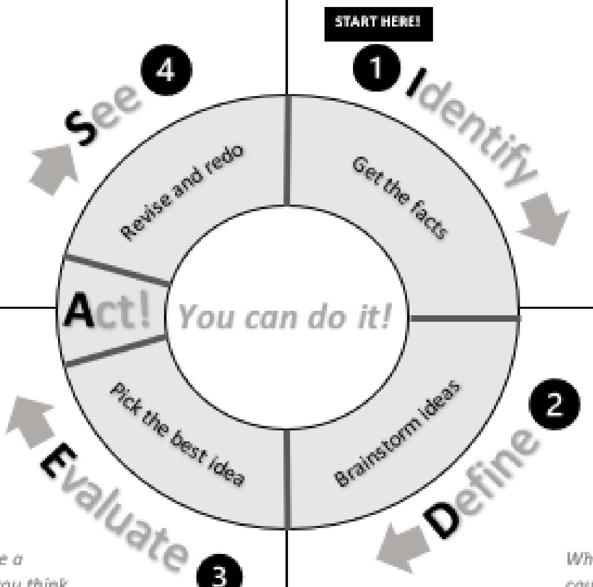

Flip over the page and make a SMART goal with the idea you think will help the most

What is one (or more!) thing you could do that might help improve the problem?

Specific
Exactly what will I do? When? Where? Who will be involved?

Measurable
How much will I do (i.e. amount, minutes)? How often?

Achievable
On a scale of 0 to 10, how confident am I that I'll be successful? If less than 7, how can I adjust my plan so that I'll be successful?

Relevant
Why is doing this important to me?

Time-bound
When will I make my first step? How long will I try this for before reviewing and updating my plan?

The biggest things that might keep me from success:

What I can try to overcome them:

SMART goal

Starting on ____________ *(day), I will* ______________________________________
____ *(what/how much)*
____ *(where/when/how often).*
I will try this until ________________________ *(day) before reviewing and updating my goal.*

Overlap Phase (Session 5) – Sick day management handout

Sick day self-care

When you get sick, your body releases hormones that raise your blood glucose levels, and you may struggle to eat or drink as much as usual. ***Both of these situations make it harder to keep your blood glucose stable.***

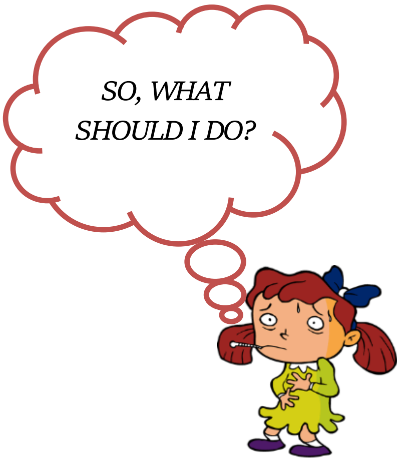

- Continue taking your insulin as usual
- Test your blood sugar every 4 hours and keep track of results
- Drink extra water to prevent dehydration. Drink ½ to 2/3 cup of water every half hour. If you have trouble keeping water down, have small sips every 15 minutes.
- Try to eat as you normally would
- Start treatment for your underlying illness, including taking medications you are prescribed
- Weigh yourself every day. Losing weight without trying is a sign of high blood sugar!
- Check your temperature every morning

If my blood glucose is higher than 180 mg/dl I should…

- Drink 8 ounces of water every hour
- Test for ketones twice every 24 hours
- Consider taking extra insulin (*see next page for table!*)

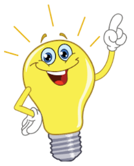

Remember to measure your ketones *regardless of your blood glucose level* if you have any of the following symptoms:

- Frequent trips to bathroom
- Nausea/vomiting/abdominal pain
- Fatigue
- Confusion or loss of consciousness

If my blood glucose is lower than 70 mg/dl I should…

Step 1.

- Take fast-acting sugar (15 grams carbohydrate)
- 3 teaspoon glucose powder dissolved in water
- ½ cup juice or soda
- 2-6 sugar candies
- 3 glucose tablets
- 1 tablespoon honey or sugar

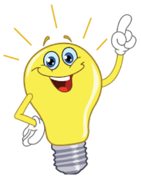

Step 2.

Wait 15 minutes then recheck blood sugar

Step 3.

If blood sugar is still below 70 mg/dl, repeat Step 1 and Step 2 until blood sugar reaches 70 mg/dl or more

Step 4.

Eat a meal or snack within 30-45 minutes

Many people tend to want to eat as much as they can when their blood glucose is low, but this can cause blood sugar to shoot way up. Follow the 15-15 Rule to avoid this!

- ✓ Check blood sugar before driving!
- ✓ Do NOT drive until 100 mg/dl

- ✓ Check blood sugar before physical activity!
- ✓ Do NOT exercise
  - until blood sugar >100 mg/dl
  - if blood sugar >250 mg/dL *and* ketones are positive

When do I know if my insulin needs are different than usual?

**Blood ketones**	**Urine ketones**	**How much insulin should I take?**		
		<180 mg/dL	180-250 mg/dL	>250 mg/dL
Less than 0.6 mmol	0 or trace	Normal dose	Normal dose	Extra 5% of total daily dose
**Mild**<br>0.6 – 1.0 mmol	Minimum	Normal dose<br>+ 1 cup of soda/juice	5% extra	5-10% extra
**Moderate**<br>1.0-1.5 mmol	Medium	Normal dose<br>+ 1 cup of soda/juice	5-10% extra	10% extra
**Severe**<br>More than 1.5 mmol	Moderate to high	5% extra	10% extra	10-20% extra

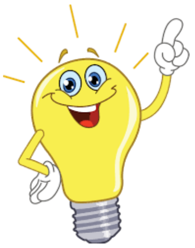

If ketones persist >1.0 mmol after you try to treat yourself on your own, contact your doctor!

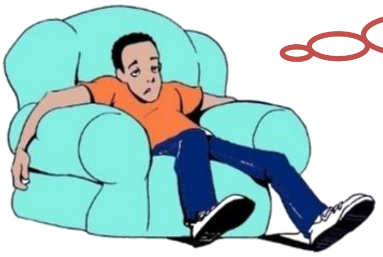

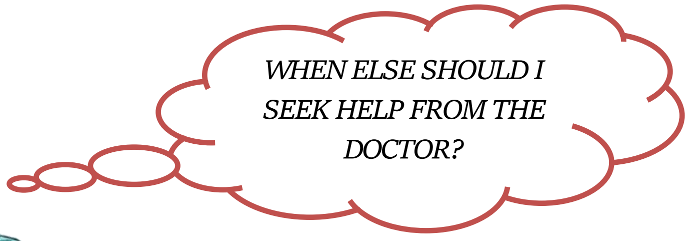

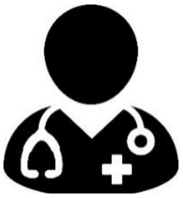

- You cannot keep liquids down for more than 4 hours
- You lose 2 kg/5 pounds or more during illness
- You feel too sick to eat normally for more than 24 hours
- You are vomiting or have diarrhea for more than 6 hours
- You have a fever over 38 ° C/ 101° F for 24 hours
- You had blood glucose lower than 70 mg/dl and you needed help from someone
- else because you were not alert enough
- You had blood glucose lower than 70 mg/dl two or more times in one week
- You had blood glucose lower than 60 mg/dl and you are not sure why
- You feel sleepy and can't think clearly

Overlap Phase (Session 5) – Insulin dose adjustment handout

Insulin Dose Adjustments

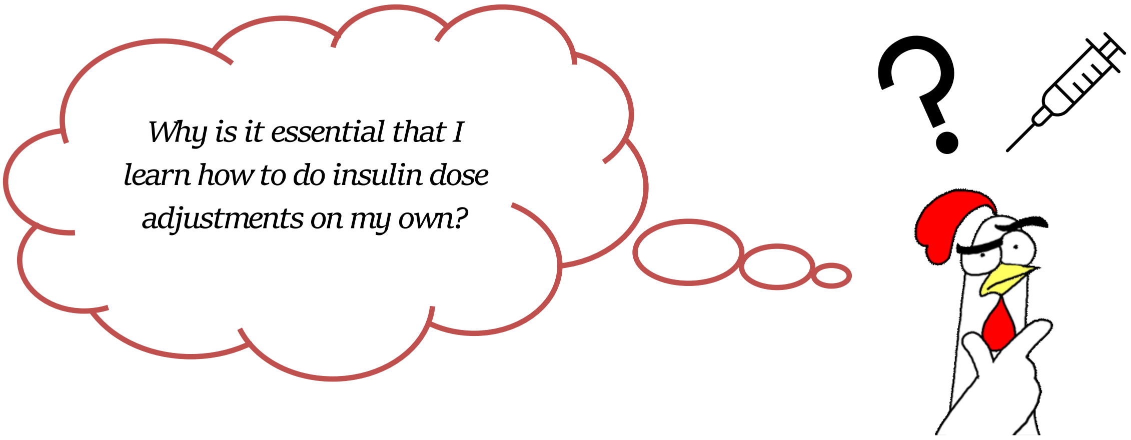

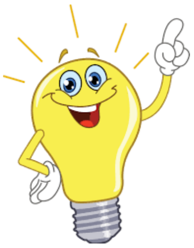

Your schedule as an adult is more dynamic than it was as a child. Your opportunities to eat and exercise are more likely to vary on a day-to-day or week-to-week basis, making your insulin needs less predictable than they were when you were a young child going to school.

As an adult, you will frequently be in situations where your parents, doctors, or other support are not there to assist you, so you must be able to do this **independently** to meet blood sugar targets now and into the future, which will help you live long and prevent diabetes complications.

When should I check my blood sugar pre-meal and post-meal?

Pre-meal:

- 30 minutes before the first bite for regular insulin (i.e., Actrapid, Humisulin R, Wosulin R)
- 5-10 minutes before the first bite for analog insulin (i.e., Aspart, Lispro, Apidra, Fiasp)

Post-meal: 2 hours after the first bite

What are my blood sugar targets that help me know whether I need an insulin dose?

Pre-meal: <120-130

Meal-related excursion: ≤ 40

Post-meal: <160-180

After dinner – fasting glucose: >40

When do I know I should adjust my basal vs. bolus insulin?

Remember that **basal** insulin acts as a more of a constant supply of insulin to help with pre-meal blood sugar levels, whereas **bolus** insulin has a shorter effect on blood sugar and so helps stabilize blood after meal blood sugar levels.

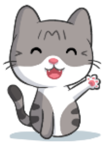

How I know that I should adjust my <u>basal</u> insulin:

I pay attention to my fasting blood sugar values…

**Fasting blood sugar**	**3 am blood sugar**	**Action**
>130-140 mg%	>100 mg%	Increase night time basal insulin dose
>130-140 mg%	70-100 mg%	Reinforce bedtime snack, consider decreasing dose of basal insulin
>130-140 mg%	<70 mg%	Nocturnal hypoglycemia related fasting hyperglycemia. Ensure bedtime snack, and decrease dose of basal insulin

How I know that I should adjust my <u>bolus</u> insulin:

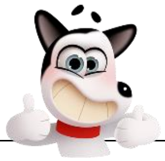

I pay attention to my pre-meal and post-meal blood sugar values…

**Pre-meal blood sugar**	**Post-meal blood sugar**	**Action**
110 mg%	140 mg%	Increment is within 40 mg%. Continue same dose.
110 mg%	180 mg%	Is the gap between insulin and meal adequate?<br>Was diet different from other days-extra carbs or fast acting carbs?<br>If no to above and consistent pattern, increase dose of pre-meal insulin
110 mg%	90 mg%	Did you eat properly?<br>Did you vomit or had loose stools after eating?<br>Did you accidentally inject higher dose of insulin, used wrong syringe (100 instead of 40 IU/ml for 40 IU/ml vial) or inject more than half hour before meal?<br>If no to above and consistent pattern, decrease dose of pre-meal insulin

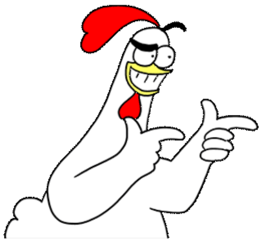
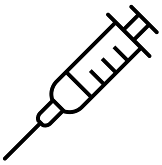
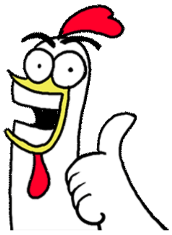

Overlap Phase (Session 5) – Relationships and Type 1 Diabetes handout

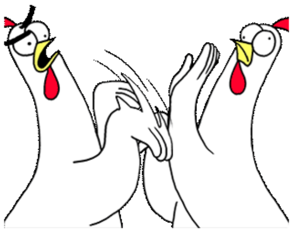

Did you know that **arguments** about diabetes management or **negative conversations** about diabetes are very common between people with type 1 diabetes and their close ones (family, friends, roommates)?

This conflict occurs between people with type 1 diabetes and their close ones even if they do not live in the same household! You might not be surprised that people who experience more of this conflict have a harder time taking care of their diabetes.

Sometimes conflict happens because it is not clear who should be taking more responsibility for your diabetes: as an emerging adult, you are being told you should be taking on most or all of your diabetes care, but for years your family might have helped with most or all the diabetes tasks.

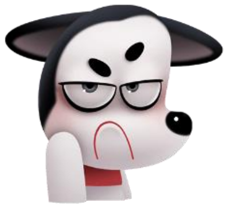

You become angry when you feel your family is "baby-ing" you when they help with your diabetes tasks or when they remind you about them.

Other times, your family might become angry and scold you that you are not taking care of certain diabetes tasks, but maybe you have never been taught how because they always took care of those things in the past.

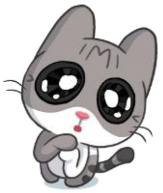

Sometimes conflict happens because family or other close relationships shame you (i.e., for unstable blood sugar), or make you feel bad about having diabetes. They may scold you because you forget, or in some cases, decide not to take care of yourself the way you have been counselled.

Because they misunderstand type 1 diabetes, they might criticize your behavior or give you incorrect advice that goes against what the doctor, educators, and dieticians have told you.

> You might not be surprised when I tell you that people with type 1 diabetes who figure out ways to get in fewer conflicts about diabetes with their close ones and set a clear plan about their own role and others' roles in their diabetes management report happier relationships, better health, and an overall happier life?

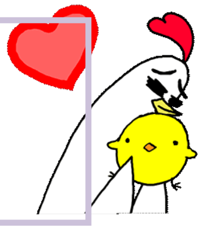

Sounds good, doesn't it? *But where do I start?*

Let's work on it together!

Overlap Phase (Session 5) – Self Care and Support Plan handout

Diabetes Self-Care and Support Plan

My **current** responsibilities for taking care of diabetes:	What others **currently** do for me related to my diabetes:
	Write **who** (i.e., mother) and **what** (i.e., inject insulin):

My responsibilities once I graduate to adult care:	What others will do once I graduate to adult care:
	Write **who** and **what** :

The one thing I will start working on (write **what, where**, and **when** you will start and achieve this goal):

The help I need to achieve this first goal:

Ways family/friends and I talk about my diabetes (or situations related to my diabetes) that causes a conflict between us:

Who	What happens	Solutions

Now let's brainstorm some strategies to prevent these conflicts!

Overlap Phase (Session 6) – Transfer proforma

Personal details

Name of the patient	
Age	
Sex	
UHID No	
Address	
E-mail	
Contact Number(s)	

Clinical details:

1. Age of diagnosis	
2. Duration of diabetes	
3. Current insulin regimen and dosage	
4. History of hospitalization	1. Yes ☐ 2. No ☐
**(If yes)**	
4.1 Number of times admitted	☐
4.2 Episodes of DKA	Yes ☐ No ☐ / If yes, specify number:
4.3 Episodes of hypoglycemia	Yes ☐ No ☐ / If yes, specify number:
5. Complications	
5.1 Retinopathy	Yes ☐ No ☐
5.2 Nephropathy	Yes ☐ No ☐
5.3 Neuropathy	Yes ☐ No ☐
5.4 If yes, details of complications and treatment	
6. Comorbidities (if any)<br>6.1 Tuberculosis<br>6.2 Invasive Fungal Infections<br>6.3 Celiac Disease	

6.4 Thyroid Disease<br>6.5 Other<br>6.6 Details of the treatment	
7. Remarks:<br>(Strengths and challenges for self-management)<br><br>7.1 Knowledge<br>7.2 Skills<br>7.3 Motivation<br>7.4 Parents/Family<br>7.5 Other social/ psychological/economic	
8. Challenges for self-management (according to patient)	
9. Additional Remarks<br>(if any)	

Name of paediatric healthcare provider: Date:

Signature:

Name of paediatric diabetes educator: Date:

Signature:

Name of the patient Date:

Signature:

Name of adult healthcare provider: Date:

Signature:
